# A multi-species model for goose management: Competition and facilitation drive space use of foraging geese

**DOI:** 10.1007/s13280-025-02206-9

**Published:** 2025-06-12

**Authors:** Monique de Jager, Nelleke H. Buitendijk, J. M. Hans Baveco, Menno Hornman, Helmut Kruckenberg, Andrea Kölzsch, Jesper Madsen, Sander Moonen, Kees H. T. Schreven, Bart A. Nolet

**Affiliations:** 1 Quantitative Biodiversity Dynamics, Department of Biology, Utrecht University, Budapestlaan 17, 3584 CD Utrecht, The Netherlands; 2 Department of Animal Ecology, Netherlands Institute of Ecology (NIOO-KNAW), Droevendaalsesteeg 10, 6708 PB Wageningen, The Netherlands; 3 Fauna Management Unit Noord-Holland, Spaarne 13, 2011 CD Haarlem, The Netherlands; 4 Environmental Sciences Group, Wageningen University and Research, Postbus 47, 6700 AA Wageningen, The Netherlands; 5 Dutch Centre For Field Ornithology (Sovon), Toernooiveld 1, 6525 ED Nijmegen, The Netherlands; 6 Institute for Waterbird and Wetlands Research (IWWR) Germany e.V., Am Steigbügel 3, 27283 Verden (Aller), Lower Saxony Germany; 7 Max Planck Institute of Animal Behavior, Am Obstberg 1, 78315 Radolfzell, Germany; 8 Ecology Department, Radboud Institute for Biological and Environmental Sciences (RIBES), Radboud University, Heyendaalseweg 135, 6525 AJ Nijmegen, The Netherlands; 9 Wageningen Environmental Reserach (WEnR), Droevendaalsesteeg 3a, 6708 PB Wageningen, The Netherlands; 10 Institute for Avian Research, An Der Vogelwarte 21, 26386 Wilhelmshaven, Germany; 11 Department of Theoretical and Computational Ecology, Institute for Biodiversity and Ecosystem Dynamics, University of Amsterdam, Science Park 904, 1098 XH Amsterdam, The Netherlands; 12 Department of Ecoscience, Aarhus University, C.F. Møllers Allé 8, 8000 Aarhus C, Denmark

**Keywords:** Barnacle goose, Farmer-goose conflict, Greater white-fronted goose, Greylag goose, Individual-based model, Pink-footed goose

## Abstract

Human-wildlife conflicts are generally tackled using single-species studies and ensuing management solutions, ignoring species interactions that may moderate or augment these issues. We investigate the effects of interactions between four goose species foraging in the province of Friesland, the Netherlands, on species distributions, energetics, and consequent economic costs of agricultural damages and management, using an individual-based model. In Friesland, geese are allowed to graze on (semi-)natural grasslands and selected agricultural grasslands (accommodation areas), while they are scared off other agricultural grasslands (scaring areas). We show that interspecific competition and facilitation drive spatial redistribution of goose species. With increasing population sizes, most species are forced to increase foraging in the scaring areas, leading to more scaring events and more additional foraging to compensate for energy spent on fleeing behaviour. Our modelling results underline that careful consideration should be given to how management may affect the spatial distribution of foraging geese.

**Supplementary Information:**

The online version contains supplementary material available at 10.1007/s13280-025-02206-9.

## Introduction

Due to anthropogenic changes, such as changes in land use or species management, some species have become vulnerable to extinction, while others have become highly abundant (Batt 1997; Jefferies et al. 2004; Allombert et al. 2005; Castro et al. 2005; Rotem et al. 2011; Oro et al. 2013). Such highly abundant species may come into conflict with humans when more intensely interfering with human interests (Marzano et al. 2013; Stroud et al. 2017) and may have a large impact on other species (Jefferies et al. 2006; Latham et al. 2011), either negatively, through for instance resource competition, or positively through facilitation (van der Wal et al. 2000; Arsenault and Owen-Smith 2002; Stahl et al. 2006). Depending on their nature, interactions between species may moderate or intensify human-wildlife conflicts; but species’ interactions are generally disregarded in studies and management concerning human-wildlife conflicts (e.g. Treves et al. 2004; Michalski et al. 2006; Kissui 2008; de Jager et al. 2019).

Many goose populations have been growing since hunting regulations were enforced; which, combined with increasingly abundant agricultural resources, resulted in an increased impact of geese on agricultural land (Fox and Madsen 2017). Different management approaches have been used in an attempt to reduce farmer-goose conflict (Fox et al. 2017). One of these approaches aims to influence the distribution of geese across the landscape, by scaring them away from some agricultural fields while providing safe refuge on others (accommodation areas) and in natural areas, combined with compensation payments to farmers (Kwak et al. 2008; Eythorsson et al. 2017).

The effectiveness of this management strategy may be influenced by species interactions. Both exploitative competition and facilitation have been documented in waterfowl. Larger species may shorten the grass and thereby facilitate smaller species that have their highest intake rates on short grass, while smaller waterfowl may crop the grass too short for species with bigger bills, thereby outcompeting the larger species (Rees 1990; Durant et al. 2003; Heuermann et al. 2011). If smaller species outcompete larger ones, they may push these larger species out of accommodation areas (Rees 1990; Kleijn et al. 2008; Tombre et al. 2019). Scaring efforts outside of accommodation areas would then have a disproportionate impact on the larger goose species, potentially reducing their fitness. The distribution and abundance of geese may affect yield loss, as well as management costs such as those associated with scaring geese away (scaring costs) and appraising damages inflicted by geese (appraisal costs) (de Jager et al. 2023, 2024). Buitendijk et al. (2022) show that species interactions between barnacle geese (*Branta leucopsis*), greater white-fronted geese (*Anser albifrons*), and greylag geese (*Anser anser*) affect the relationships between species abundance and yield loss; however, a mechanistic understanding of how species interactions affect total costs, including yield loss, appraisal costs, and scaring costs, is lacking.

Here, we examine how four goose species with varying body sizes and population sizes, affect each other’s spatial distribution through facilitation and competition, assuming management with scaring and accommodation areas. We subsequently investigate the emergent interaction effects of these species on yield loss, appraisal costs, and scaring costs. For this purpose, we extended an existing individual-based model (de Jager et al. 2023, 2024). With individual-based models, populations can be simulated by following individuals or—as in our case—groups of individuals (Scheffer et al. 1995). Custom-made individual-based models are optimal tools to analyse complex ecological questions (DeAngelis and Mooij 2005; MacPherson and Gras 2016; van der Vaart et al. 2016) and to address important questions in wildlife management and environmental practices (Wood et al. 2015). We adapted a model simulating barnacle geese (average initial weight 1771 g; Table 1) foraging in the province of Friesland, the Netherlands (de Jager et al. 2023), to also include the three next most prevalent goose species in Friesland: (greater) white-fronted geese (2127 g), pink-footed geese (*Anser brachyrhynchus*; 2170 g), and greylag geese (3401 g). Barnacle geese are the most abundant goose species in Friesland, with a wintering population of approximately 500 000 individuals in 2018/2019 (Hornman et al. 2021). They likely contribute most to the farmer-goose conflict in terms of yield loss on agricultural grassland in spring (Buitendijk et al. 2022). The other goose populations are (substantially) smaller (approximately 300 000 white-fronted geese, 70 000 greylag geese, and 15 000 pink-footed geese in 2018/2019; Hornman et al. 2021) and their impact on yield loss is expected to be smaller as well (Buitendijk et al. 2022). In 2023, damages to grasslands in the province of Friesland have been attributed to 17 different animal species; approximately 89% of these damages were credited to barnacle (37%), greylag (37%) and while-fronted geese (15%)^1^.

**Table 1:** Parameter values that were added to the single-species model (de Jager et al. 2023) to include four goose species. (Parameters that remained the same as in De Jager et al. 2023 are not shown in this table.)

Source	Parameter name	Parameter value			
		Barnacle goose	Greylag goose	Pink-footed goose	White-fronted goose
	Abbreviation	BAG	GLG	PFG	WFG
Baveco et al. (2011)	Flight speed (m/s)	19.0	19.2	18.8	15.0
	Field metabolic rate (J/s)	9.84	17.14	16.51	13.97
	Functional response b1 (g/m)	0.33	0.25	0.23	0.25
	Functional response b2 (g/m)	76	10	21	29
	Functional response c (s/m)	1.0	0.0	0.5	0.5
	Minimal cropping time (s)	0.48	0.80	0.59	0.59
	Maximal chewing rate (g/s)	0.02	0.08	0.04	0.03
	Max. instantaneous intake rate (g/s)	0.005	0.02	0.009	0.008
	Resting metabolic rate (J/s)	7.25	12.63	12.17	10.29
	Flight metabolic rate (J/s)	60.80	123.70	97.70	76.10
Capture data	Initial weight (g)	1771	3401	2170	2127
	Minimum weight (g)	1080	2040	1600	1400
	Daily average weight (g)	195 values, calculated per species			
	Maximal additional weight (g)	600	900	600	400
Count data	Maximum number of geese	500 000	70 000	15 000	300 000
	Flock size	1000	100	200	400
	Number of flocks	500	700	75	750
	Initial spatial distribution	61 sites	65 sites	9 sites	59 sites
	Probability to join flocks—*a*_*1*_	− 2.17	− 2.19	− 1.41	− 2.17
GPS-tracks	Roost locations	combined all species' GPS and roost count locations			
	Flight time coefficient *c*_*1*_	0.20	0.25	0.18	0.57
	Flight time coefficient *c*_*2*_	0.52	0.52	0.48	0.44
	Composite random walk—*λ*	0.08	0.116	0.052	0.05
	Composite random walk—*μ*	1.74	2.08	2.25	1.64
	Composite random walk—*x*_*max*_	200	84	200	200
	Composite random walk—*P*_*BW*_	0.32	0.46	0.4	0.18

In the province of Friesland, goose management through scaring and accommodation is implemented (Kwak et al. 2008). The total costs involved in the farmer-goose conflict depend on many factors, most of which are linked to spatial distributions of geese, and are thus potentially affected by interactions within and between species. First, costs of scaring geese out of scaring areas (i.e. agricultural grasslands where scaring is allowed) is affected by the number of flocks that need to be scared off, which in turn depends on goose population size, goose flock size, and the spatial distribution of flocks over accommodation and scaring areas (de Jager et al. 2023, 2024). Second, damage appraisal costs depend on the number of fields affected by grazing, which in turn is determined by the spatial distribution of flocks (de Jager et al. 2023, 2024). Third and most important, the appraised yield loss depends on the amount of grass being depleted, which is determined by goose foraging time and instantaneous intake rate (Baveco et al. 2011). Foraging time on agricultural grasslands is in turn affected by energetic requirements, which can differ between individuals and species due to variation in flight time (which can be majorly influenced by scaring; Belanger and Bedard 1990; Béchet et al. 2004; Nolet et al. 2016), minimal required body mass, and basal metabolic rates, amongst others. Furthermore, the spatial distribution of geese influences the foraging time on agricultural grassland; more grazing time on natural grasslands decreases foraging time on agricultural grasslands and hence lowers yield loss. The interplay between the locations of roost-sites and agricultural and natural grasslands also affects the spatial distribution of geese.

Because nature areas are close to roost-sites, we hypothesize that these areas are frequently used, and that, with increasing population sizes, the fraction of foraging time spent on grasslands in nature areas decreases as more and more geese need to forage on agricultural grasslands (De Jager et al. 2024). We also hypothesize that larger goose species with higher energetic requirements (Baveco et al. 2011) and faster intake rates (Durant et al. 2004) have the largest impact per goose on yield loss, but that smaller goose species are able to graze down the grass further (Durant et al. 2003), thereby further increasing yield loss locally. Barnacle geese, which preferably forage on shorter grass than the larger species (Durant et al. 2004), are expected to be facilitated by those larger species. They may also indirectly push the larger species to forage outside of accommodation areas, by maintaining a sward height unsuitable for the larger species (Kleijn et al. 2008).

## Materials and methods

The model is based on an existing single-species individual-based model, which simulates the spatial distribution of flocks of barnacle geese during winter foraging in the province of Friesland, the Netherlands (de Jager et al. 2023). A detailed description of the single-species model can be found in de Jager et al. (2023), and a full account of the multi-species model is given in supplementary methods A, following the ODD protocol (Grimm et al. 2006, 2020). Here, we provide a short overview of this extended model.

Flocks of 1000 barnacle geese, 100 greylag geese, 200 pink-footed geese, and 400 white-fronted geese are the agents in the model. Per species, all flocks behaved similarly, differing in their starting location only. The model runs from November 1st to May 15, with a temporal resolution of 1 h (day of the year 305–135, 4680 time-steps). The spatial scale is set as such that each patch represents 100 × 100 m (= 1 ha). The total extent of the modelled area is 70 × 70 km (= 700 × 700 patches of 1 ha). Each patch contains a patch type (− 1 for roost, 0 for other, 1 for agricultural grassland in scaring area, 2 for agricultural grassland in accommodation area, and 3 for (semi-)natural grassland in nature area). We used ‘Basisregistratie gewaspercelen’ (‘master registry of agricultural parcels’) to determine which patches are grassland. These were then divided into nature area (using ‘natuurbeheerplannen 2021’-data, 12 546 patches), accommodation area (‘ganzenfoerageergebied 2021’-data, 15 533 patches) and scaring area (remaining grassland patches, 139 324 patches; de Jager et al. 2023). Non-grassland patches were divided into roost-sites (7881 patches) and other. Using hourly GPS-points from tracked geese, we defined roost-sites as locations within a 1-km area that were visited during at least four nights (between 0:00 and 4:00 h, local time). Patch types 1, 2, and 3 also contain the variable grass height, and record the number of flock-hours that flocks have spent on the patch. Initial grass height at grassland patches was set at 0.094 m (Baveco et al. 2011).

We initialized the spatial distribution of our modelled geese based on roost count estimates collected in 2019 by the Dutch Centre for Field Ornithology (Sovon; Hornman et al. 2021). Sovon also provided monthly daytime goose counts covering the whole province. These were used to determine the total number of geese present in each month (Fig. S1), which changes with migratory arrival and departure (with a peak in January–February; Hornman et al. 2021).

Each time step, a flock follows the decision tree illustrated in Fig. S2. During daylight hours, flocks move to a selected roost, if maximum weight (median goose weight estimated from field studies for that date plus a maximal additional weight; Table 1; Fig. S3) was reached. If not, they continue foraging. Foraging patches are selected based on memory or at random, depending on memory decay rate (*λ*), maximum probability to forage on memory (*P*_max*M*_), and memorized grass heights. To limit memory size, the oldest memory is replaced by the newest one, keeping a constant memory size of 100 locations and their grass heights. A memorized patch is selected based on memory age, the expected energy gain, and the energy required to move there, making it likely that the same patch is used in multiple consecutive time-steps. When foraging randomly, a patch is selected using a composite random walk, consisting of a Brownian (exponential distribution) and a truncated Lévy (bounded Pareto distribution) walk (Kolzsch et al. 2015).

During flight to the selected patch, the flock may join a patch with other foraging geese of the same species, depending on the number of geese already present, as flocks are attracted to other flocks of the same species. After arriving at a patch, flocks choose whether to forage there, or move again, depending on grass height and maximum probability to forage at a patch (*P*_max*F*_); they prefer to forage on grass of a height that maximizes species-specific intake rate. Additionally, flocks move if a disturbance occurs, which is more likely in scaring areas. After a flock is disturbed, it flies up and across a distance of 2 km (Nolet et al. 2016) in a random direction before moving to a new foraging site. Memorized grass height for the patch at which the scaring occurred is set to zero, making a return in subsequent time-steps less likely.

As geese have been observed to forage at night (Giroux 1991; Mooij 1992; Lameris et al. 2021), we included this possibility when the flock has a lower than expected weight (Fig. S3), provided they can rest for at least eight hours, and sufficient moonlight is available. Alternatively, geese roost from sunset to sunrise. Flocks return to their previous roost-site if located within 10 km; otherwise a random roost-site is chosen, weighed by the distance to the current location.

Foraging and movement behaviour influence goose energy intake and expenditure. A flock dies when the weight of its geese falls below a species-specific minimum weight value (Table 1). Foraging behaviour also reduces grass height at grazed patches. Each day the grass grows, following Monteith (Monteith 1977), depending on temperature and solar radiation. Empirical studies have shown that grass growth rate also depends on the height of the grass and can therefore be affected by goose grazing (Buitendijk and Nolet 2023; Duettmann et al. 2023). Including this in the model would, however, extremely amplify computation time. It was taken into account through a sensitivity test (see Section *Sensitivity analysis and model validation*)*.*

As our model extends the existing barnacle goose model (de Jager et al. 2023) with three additional goose species, some parameter values were added (Table 1). A number of parameter values could be obtained from literature (such as flight speed, metabolic rates, functional responses, and intake rates; Baveco et al. 2011); others needed to be estimated using capture data (e.g. body weights), count data (e.g. flock sizes, population sizes, initial spatial distributions, and probability to join other foraging flocks), or GPS-tracking data (e.g. roost locations, flight time coefficients, and composite random walk parameters).

### Capture data

Geese were weighed during capture events for ringing using a standard method (Beer and Boyd 1962; Ebbinge et al. 2010; Koelzsch et al. 2016). We used these data to estimate the average and maximum goose weight per species per day, for the simulation period (November 1st to May 15th, Fig. S3). The initial weight of the simulated geese was set at the average weight per species observed between November 1st and November 5th (the first five simulation days). The minimum weight per species, below which a goose is expected to die, was fixed at the recorded minimum weight for that species between November 1st and May 15th.

### Count data

Goose numbers were estimated from the monthly daytime counts performed by Sovon in Friesland in 2016–2019 (Hornman et al. 2021). For each year, we summed the counts per month per goose species and used the maximum as our estimated maximum value for the number of geese per species per month (Fig. S1). Using a quadratic function per species (with the estimated maximum number as highest value), we estimated the number of geese of that species in Friesland per simulation day.

Using recorded flock sizes and similar methods as in de Jager et al. (2023), we fixed model flock sizes around the 20-percentile number of geese per actual flock (Fig. S4, Table 1). The number of geese observed foraging together was also used to estimate the probability that a flock will join a group of conspecifics foraging on a patch.

We used the same methods as in de Jager et al. (2023) to initialize the spatial distributions of the goose species across the roost patches in our extended model, in accordance with their relative roost count abundances (Hornman et al. 2021).

### GPS-tracking data

We used GPS-tracks from barnacle geese (*n* = 97), greylag geese (*n* = 94), pink-footed geese (*n* = 132), and white-fronted geese (*n* = 149), collected in the period 2016–2020 (Table S1). For the months November to May (day of the year 305–135), we selected GPS-points within the boundaries of Friesland (52.82 < latitude < 53.52; 5.35 < longitude < 6.35). As too few data points were available for greylag geese in Friesland, we extended the spatial boundaries for this species to also include the neighbouring province Groningen (52.8 < latitude < 53.8; 5.3 < longitude < 7.3). Because these two provinces have similar landscapes, we assume that the behaviour of greylag geese residing in Groningen is similar to the behaviour of greylag geese in Friesland.

Using the GPS-locations between midnight and 4 am, we located the roost-site patches that can be used in our model. We regarded any location (i.e. a 100 × 100 m grid cell) that has been visited four or more times by a goose species as a roost patch. We combined all observed roost patches as roosting locations that can be used by all four species, resulting in 10 188 roost patches (Fig. S5).

Geese usually do not fly directly to a foraging patch, but circle a patch several times before landing. As in De Jager et al. (2023), we used GPS-tracking data to estimate flight duration per flight distance, per species (Fig. S6). We assumed that a goose was flying when the recorded velocity exceeded 12 m/s, and we only used consecutive GPS-locations if the time interval was less than or equal to 15 min. We estimated a species’ flight duration as ${T}_{v}= {e}^{S}\cdot \frac{d\cdot {L}_{\text{patch}}}{v}$, where *d* is the flight distance (in patch units), *L*_patch_ the length of a patch (in m/patch), *v* is the flight speed (in m/s) (Baveco et al. 2011), and *S* a logarithmic sigmoid function that significantly increases the modelled relation between flight distance and duration in all four species:

1 $$S=\frac{10}{1+ {c}_{1}\cdot (d\cdot {L}_{\text{patch}}\cdot {c}_{3}{)}^{{c}_{2}}},$$

where *c*_*1*_ and *c*_*2*_ are species-specific coefficients (Table 1), and *c*_*3*_ (*c*_*3*_ = 1 m^−1^) makes the function unitless.

When foraging randomly instead of on memory, a random foraging site is selected using a composite random walk, consisting of a Brownian walk (exponential distribution) and a truncated Levy walk (bounded Pareto distribution; Fig. S7). Per species, we created a composite random walk, in which *P*_*x*_ is the probability that a flight length *x* (in hm) is drawn from the frequency distribution:

2 $${P}_{x}= {P}_{\text{BW}}{e}^{-\lambda x}+(1- {P}_{\text{BW}})(1-\frac{1- {x}_{\text{min}}^{\mu -1}{x}^{1-\mu }}{1-\left(\frac{{x}_{\text{min}}}{{x}_{\text{max}}}{)}^{\mu -1}\right)},$$

where *x*_min_ is the minimum flight distance (*x*_min_ = 1 hm) and *x*_max_ is the maximum flight length. We used the distances moved per hour by GPS-tracked geese (de Jager et al. 2023) to estimate the parameters *μ*, *λ*, *x*_max_, and *P*_BW_. The parameter *P*_BW_ gives the probability that a flight length is drawn from an exponential distribution rather than a bounded pareto distribution. Parameters per species were estimated by maximum likelihood estimation of the calculated inverse cumulative frequency distribution (ICFD) on the observed ICFD of distances moved per hour. For this parameter estimation procedure, we used the mle2 function from the bbmle package in R.

### Sensitivity analysis and model validation

We evaluated whether the chosen model parameters and rules provided reasonable results during the model validation phase by running additional simulations with other than the default settings. We also compared some model results to real world observations. While we modelled grass growth following Monteith (1977), in reality, grass growth depends on initial grass height and time of year (Buitendijk and Nolet 2023). To examine the validity of our simpler model assumption of height-independent grass growth, we compared simulation results between the simpler model and a more refined (and computationally more extensive) model (Supplementary methods A: 7.11). Additionally, we compared simulation results between models differing in flock sizes, as flock size may affect flock survival and thereby may influence model outcomes. Next to the flock sizes used by default (BAG = 1000, GLG = 100, PFG = 200, and WFG = 400), we ran simulations with half of these flock sizes and doubled flock sizes. We ran simulations with all combinations of 3 different population sizes per species (= 81 simulations).

We furthermore verified that the model results in reasonable and consistent goose foraging behaviour for all four species by running additional simulations with the current population sizes. The decisions made in the model are at flock- and patch-level, and the relations at the larger spatial scales are emergent properties thereof. Using the results of 100 simulations, we examined the spatial distribution of the geese across the different parts of Friesland as well as across nature, accommodation, and scaring areas. Using the results of 10 additional simulations, we inspected how well goose body mass in our simulations resembled that of actual geese.

### Model simulations

To evaluate the influence of different population sizes, we ran simulations with 11^4^ = 14 641 different combinations of the four population sizes, using a full orthogonal sampling of the 4-dimensional parameter combination matrix. Population sizes ranged between 0 and 200% of current wintering population sizes in Friesland (Table 1), using 11 different (evenly-spaced) population sizes per species. After each simulation, we recorded the average goose pressure (summed goose h ha^−1^) per grassland type (scaring, accommodation, or nature area) per species, average grass height per grassland type, the number of patches affected by geese (i.e. having been foraged upon by at least one flock) per grassland type and goose species, and total yield loss. As in de Jager et al. (2023) and (2024), yield loss (in cost per ha) was calculated from the difference in grass height (in cm) between unaffected grasslands and the focal patch, by multiplying this height difference with 150 (dry matter weight in kg of 1 cm grass across 1 ha) and € 0.25 (monetary value of 1 kg dry grass). We furthermore calculated additional foraging per species, which is the extra goose pressure (at all grassland types, in summed goose h ha^−1^) that geese forage on top of the minimum goose pressure observed during the simulations. Such additional feeding is required when, for example, geese have spent much energy on flying and compensate for this energy expenditure.

### Data analysis

We estimated the relative importance of changes in the four species’ population sizes on each species (i) fraction of the focal species’ foraging in agricultural areas (which includes both scaring and accommodation areas, but not nature areas), (ii) fraction of the focal species’ foraging in scaring areas, (iii) focal species’ goose pressure per goose on agricultural grasslands, and (iv) total goose pressure on all grasslands, including those in nature areas. In each case, we generated a linear model (*y* ~ *β*_*0*_ + *β*_*1*_*N*_*BAG*_ + *β*_*2*_*N*_*GLG*_ + *β*_*3*_*N*_*PFG*_ + *β*_*4*_*N*_*WFG*_ + *ε*); the coefficient estimates (β_1_–β_4_) were normalized through division by the maximum deviation from 0. *N*_*BAG*_, *N*_*GLG*_, *N*_*PFG*_, and *N*_*WFG*_ represent the numbers of barnacle geese, greylag geese, pink-footed geese, and white-fronted geese, respectively. These normalized coefficient estimates indicate the relative importance of changes in the four species’ population sizes, ranging between − 1 and 1, with − 1 being the most important negative impact and 1 being the largest positive impact. Goose pressure per goose was calculated as the average number of hours per day a goose of the focal species has spent on agricultural grasslands. Total goose pressure per goose is the average number of hours per day a goose of the focal species has spent foraging in both agricultural and nature areas. Similar to the data analyses presented above, we furthermore estimated the relative importance of changes in the four species’ population sizes on (i) yield loss, (ii) the number of patches affected by foraging geese in scaring areas, and (iii) the number of scaring events.

## Results

### Sensitivity analysis and validation

Adding grass height dependent grass growth (GHDGG) to the model instead of using the simpler grass growth calculations based solely on temperature and solar radiation had little to no effect on the qualitative outcomes of our study (Figs. S8, S9). The fraction of geese on agricultural grasslands, the fraction of geese foraging in scaring areas, average goose pressure per goose, and total goose pressure per goose were comparable with those of the simpler model for all four species (Fig. S8). Exceptions were the somewhat higher goose pressure per greylag goose in the simulations with GHDGG, and the, respectively, lower and higher fractions of pink-footed and white-fronted geese in scaring areas. Yield loss and total costs were lower for all species in case of GHDGG (Fig. S9), which is to be expected. However, this quantitative difference in results did not affect the relations between the species and the general conclusions that we can draw from this modelling study.

Similarly, changing the flock sizes of the four goose species did not affect the qualitative results of our study (Figs. S8, S9). While flock size had its effects on the goose pressure and total goose pressure per goose (Fig. S8), the relations between the different goose species remained basically the same. Due to the increase in the number of flocks, flock sizes negatively affected the percentage of scaring patches visited by geese, the number of scaring events, and the total costs (Fig. S9).

**Fig. 1:**
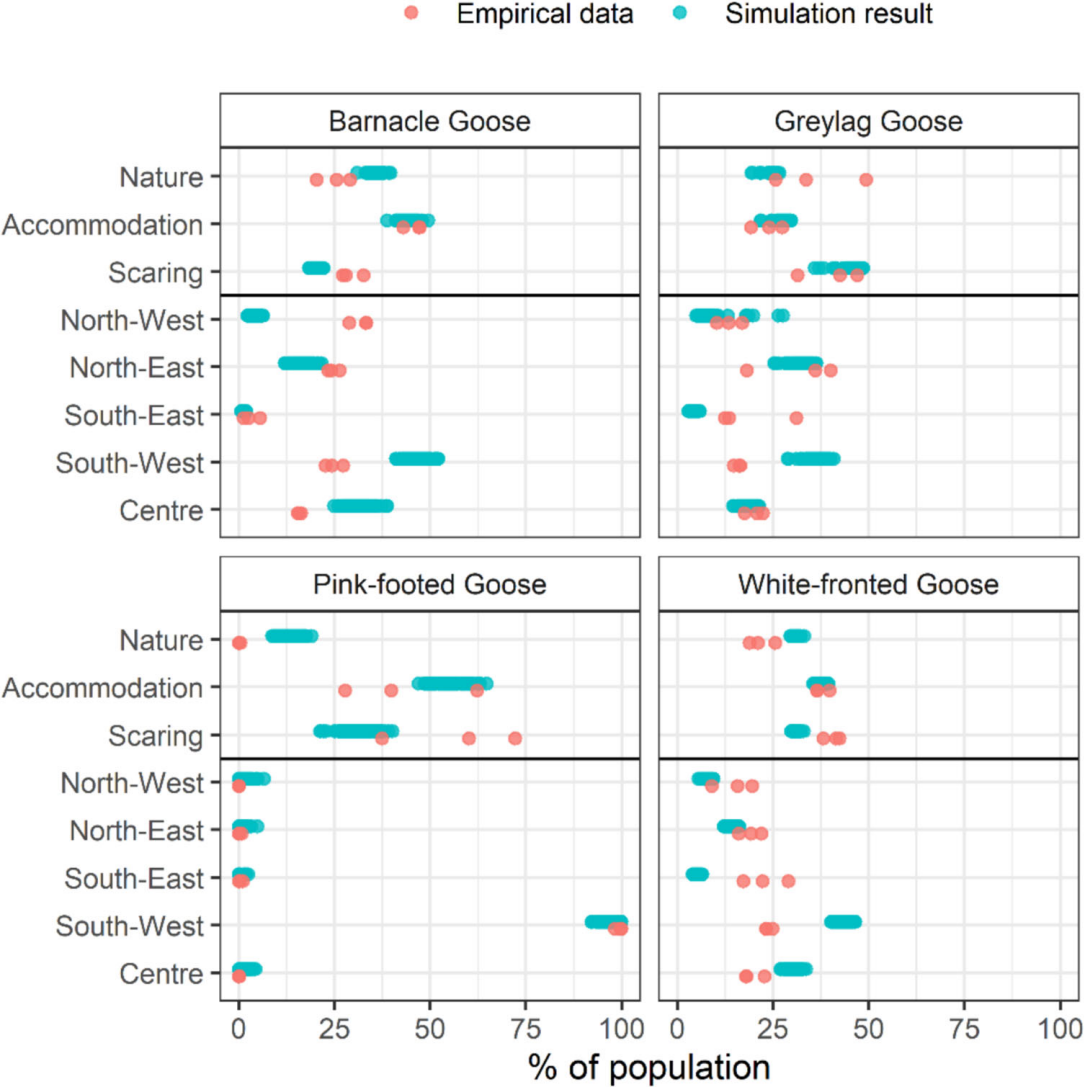
Comparison of simulation output (blue) and empirical data (salmon) for the spatial distributions across nature, accommodation, and scaring areas as well as across different regions (North-West, North-East, South-East, South-West, and the Centre area of Friesland) of barnacle, greylag, pink-footed, and white-fronted geese. Empirical count data for the seasons 2016/2017, 2017/2018, and 2018/2019 was derived from Hornman et al. (2021). Both count data and simulation output were transformed to percentages of geese of that species present in that area

**Fig. 2:**
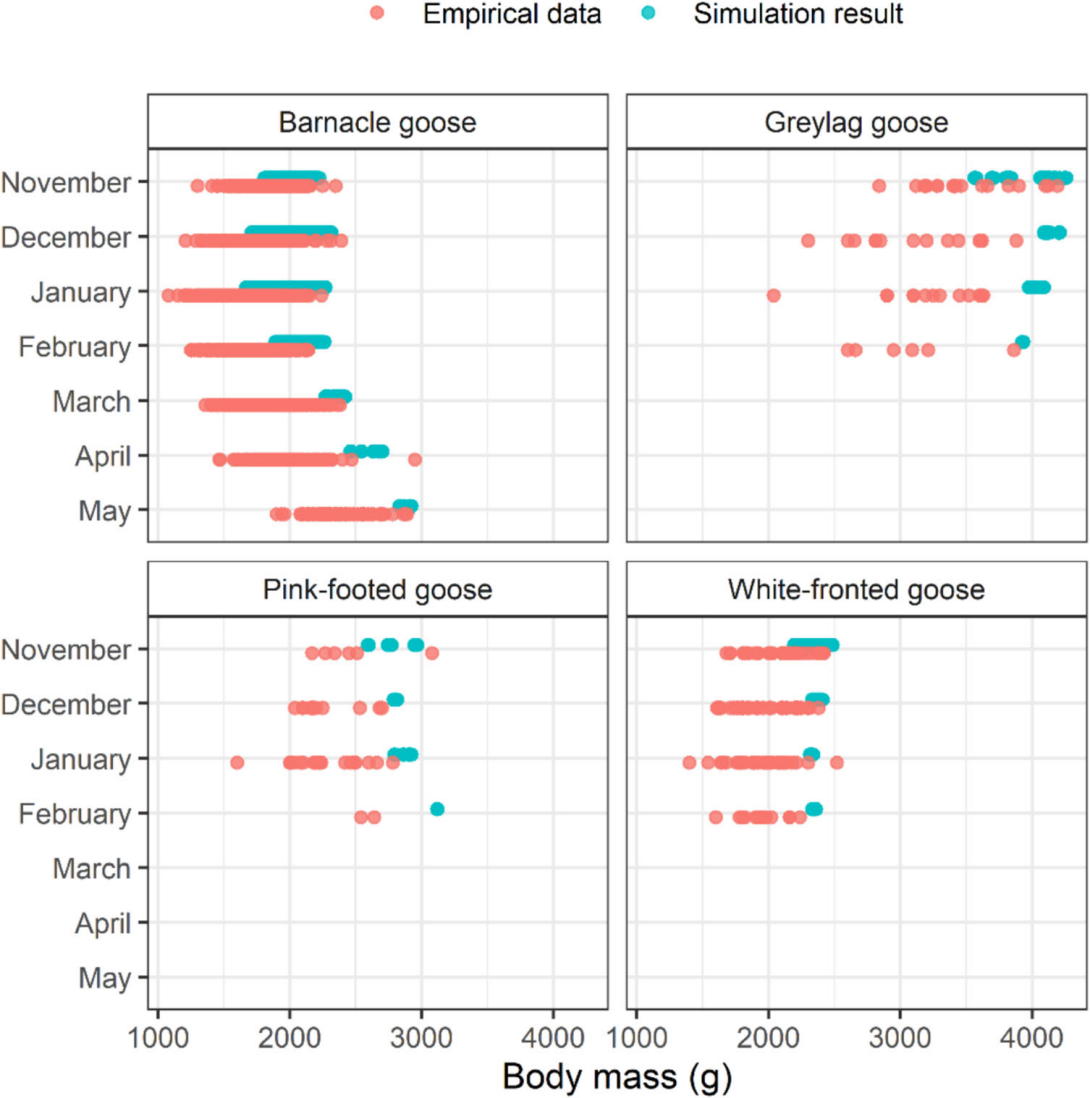
Comparison of simulation output (blue) and empirical data (salmon) for body mass per species

Overall, simulation results represent empirical patterns in spatial distributions quite well (Fig. 1). The percentage of geese residing in certain areas during model simulations overlapped with that of at least one of the three empirical observation seasons in 20 of the 32 cases. The largest difference occurred for barnacle geese, where real geese were much more prevalent in the north-western area of Friesland compared to the model results. In contrast, there were fewer empirically observed barnacle geese in the south-western area as opposed to the simulation output. For both greylag and white-fronted geese, fewer geese were estimated by our model to forage in the south-western part of Friesland compared to the empirical data, while modelled geese resided more frequently in the south-western part. Modelled pink-footed geese foraged more in nature areas than expected from empirical count data (Hornman et al. 2021). Goose body mass during model simulations was slightly on the high side compared to empirical data, especially for greylag and pink-footed geese (Fig. 2).

### Effects of population sizes on species’ distributions

**Fig. 3:**
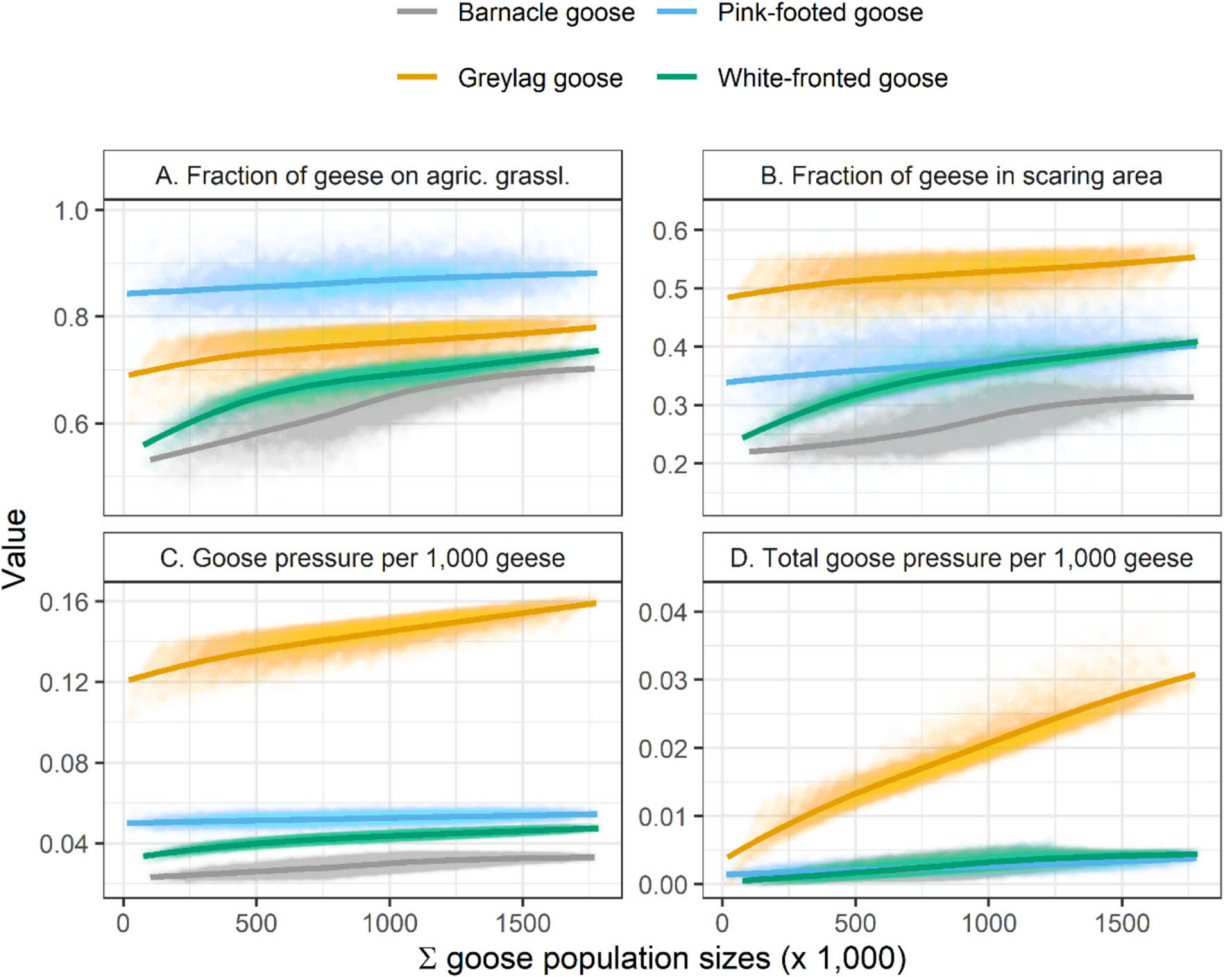
The relation between the sum of all four goose population sizes per simulation and the fraction of geese of the focal goose species (indicated by colour) foraging on agricultural grassland rather than in nature area (**A**), the fraction of geese of the focal species foraging in the scaring area (**B**), average goose pressure per 1,000 geese of the focal species (in goose h ha^−1^ day^−1^, **C**), and total goose pressure per 1,000 geese of the focal species (also in goose h ha^−1^ day^−1^, **D**). Goose pressure in **C** and **D** is calculated for agricultural grassland only. Lines show the local polynomial regression lines between the data points per species

For all four species, our model predicts that the fraction of geese foraging on agricultural rather than on natural grasslands increased with rising goose numbers (Fig. 3A). The fraction of pink-footed geese on agricultural grassland was substantially higher than that of the other three species (Fig. 3A). For all species, the fraction of geese foraging on agricultural grasslands was most strongly impacted by the species’ own population size (Table 2a), but, in contrast to the other species, the fraction of pink-footed geese on agricultural grasslands decreased with an increase in the species’ own population size.

**Table 2:** Relative importance of the variables and adjusted R^2^ of the linear regression models, where we examined the effects of barnacle (BAG), greylag (GLG), pink-footed (PFG), and white-fronted (WFG) population sizes (N_BAG_, N_GLG_, N_PFG_, and N_WFG_, respectively) on species-specific (a) fractions of foraging time spent on agricultural grassland, (b) fractions of foraging time spent in the scaring area, (c) goose pressures per goose, and (d) total foraging per goose. Negative values represent negative effects of population size on the dependent variable; for example, the fraction of barnacle geese foraging in scaring area is negatively related to population sizes of the other three species

	*R*^2^	Variable importance			
		*N*_BAG_	*N*_GLG_	*N*_PFG_	*N*_WFG_
*a. Fraction foraging on agricultural grasslands*					
BAG	0.803	**1.00**	0.00	0.34	0.13
GLG	0.802	0.05	**1.00**	0.00	0.04
PFG	0.068	0.03	0.01	− **1.00**	0.02
WFG	0.769	0.37	0.44	− 0.14	**1.00**
*b. Fraction foraging in scaring area*					
BAG	0.925	**1.00**	− 0.14	− 0.27	− 0.22
GLG	0.899	0.05	**1.00**	0.03	0.04
PFG	0.107	0.04	0.14	**1.00**	0.04
WFG	0.915	0.99	0.70	0.30	**1.00**
*c. Goose pressure per goose (on agricultural grassland)*					
BAG	0.931	**1.00**	− 0.18	0.03	− 0.06
GLG	0.939	0.14	**1.00**	0.06	0.13
PFG	0.248	0.17	0.26	− **1.00**	0.21
WFG	0.909	0.87	0.87	0.20	**1.00**
*d. Total goose pressure per goose*					
BAG	0.938	**1.00**	− 0.58	− 0.32	− 0.29
GLG	0.902	**1.00**	− 0.06	0.46	0.95
PFG	0.483	0.13	0.30	**1.00**	0.26
WFG	0.914	0.87	**1.00**	0.58	0.04

The fraction of geese foraging in scaring areas instead of in accommodation and nature areas also increased with the total number of geese simulated in the model (Fig. 3B). In scaring areas, greylag geese were by far the most dominant species, followed by pink-footed geese. The fraction of barnacle geese in scaring areas was much lower than that of the other species, regardless of the total number of simulated geese (Fig. 3B). For all species, the fraction of their population foraging in scaring areas was mostly impacted positively by the species’ own population size (Table 2b). The fraction of barnacle geese in scaring areas was negatively related to greylag, pink-footed, and greater white-fronted goose population sizes. A larger portion of white-fronted geese foraged in scaring areas with larger population sizes of barnacle geese (Table 2b).

The goose pressure per greylag goose (on agricultural grassland only) was substantially higher than that of the other three species, regardless of the total number of simulated geese (Fig. 3C). For all species, the model predicted that goose pressure per goose increased with the total number of simulated geese, corresponding to a larger fraction of the populations foraging on agricultural grassland (Fig. 3A). Goose pressure per goose was most impacted by each species’ own population size (Table 2c). White-fronted goose pressure per goose was also strongly positively affected by barnacle and greylag goose population sizes. In contrast to the other species, goose pressure per pink-footed goose was negatively related to the species’ own population size (Table 2c).

The total goose pressure per goose by greylag geese (on all agricultural and natural grasslands) increased more strongly with the overall population size than for barnacle, pink-footed, and white-fronted geese (Fig. 3D), suggesting a larger increase in grass uptake for this species. Total goose pressures per barnacle and pink-footed goose were mostly affected by the species’ own population sizes (Table 2d). Interestingly, for barnacle geese the total goose pressure per goose decreased with increasing numbers of greylag, pink-footed, and white-fronted geese. Total goose pressure per greylag goose increased with growing barnacle and white-fronted goose population sizes. Similarly, total goose pressure per white-fronted goose had a strong positive relation to barnacle and greylag goose population sizes (Table 2d).

### Effects of population sizes on yield loss, number of scaring events, % scaring patches affected by geese, and total costs

**Fig. 4:**
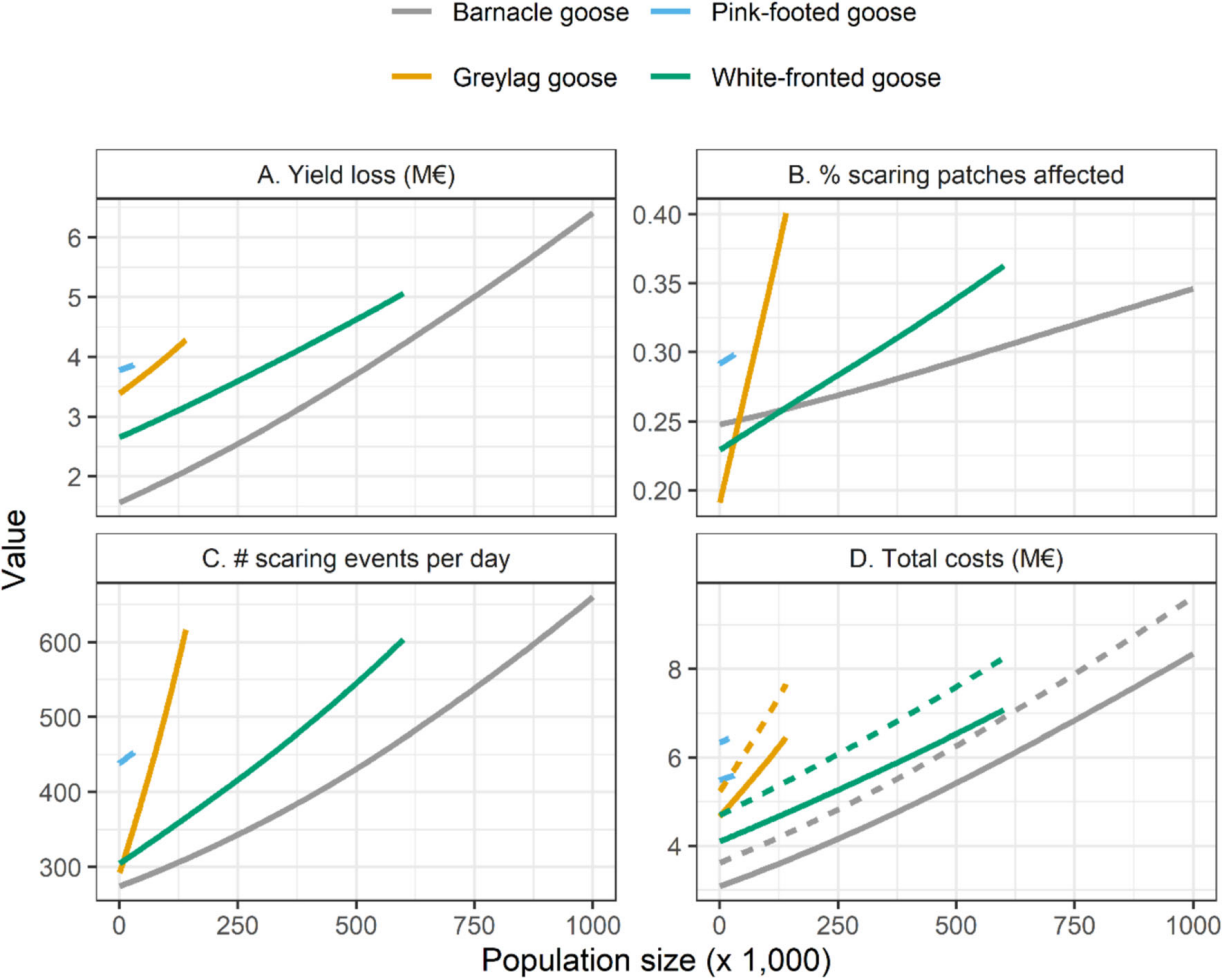
**A** Yield loss in M€, **B** percentage of agricultural patches affected by geese, **C** the number of scaring events per day, and **D** total costs without scaring costs (solid lines) and with scaring costs of €10 per scaring event (dashed lines), per species’ population size. Colours indicate the four different goose species. Lines show the local polynomial regression lines between data points

Yield loss increased with the population sizes of the four goose species (linear regression model: yield loss ~ *N*_BAG_ + *N*_GLG_ + *N*_PFG_ + *N*_WFG_; *R*^2^ = 0.996; Fig. 4A). Increasing the greylag goose population size had the largest impact on yield loss, followed by changing the population size of barnacle geese. Changes in pink-footed goose population size had the smallest effect on yield loss.

Besides yield loss, total costs of geese foraging on agricultural grasslands also depend on the number of agricultural patches visited by geese in scaring areas and the number of scaring events. The number of agricultural patches in the scaring area that have been visited by geese positively related with the population sizes of the four species (*R*^2^ = 0.974; Fig. 4B). Increasing the greylag goose population size had the strongest effect on the number of visited patches, while changes in barnacle goose population size had the smallest effect (Fig. 4B). The number of scaring events occurring during a simulation also increased with all population sizes (*R*^2^ = 0.992; Fig. 4C). The strongest effect was predicted for changes in greylag goose population sizes, while increasing the barnacle goose population size had the smallest effect on the number of scaring events. Because the number of scaring events increased rapidly with increasing greylag goose population size, the slope of the relation between total economic costs (including yield loss costs, appraisal costs, and scaring costs) and greylag goose population size increased with rising costs per scaring event (Fig. 4D).

## Discussion

Our model predicts that the fraction of the goose populations foraging on agricultural grassland increases with population size, leading to higher grazing pressures and more agricultural damage per goose. For most species, the grasslands in natural areas are likely to become saturated first, which fits with previous studies (Baveco et al. 2011; Si et al. 2011). Increasing population sizes past the capacity within nature areas may push a large part of the populations to forage on agricultural land. With higher numbers in the scaring areas, total costs per goose—including appraisal and scaring costs—also increases with population size. Added to this is the increased total amount of foraging that geese need to do as a consequence of scaring, resulting in higher overall costs (Nolet et al. 2016).

One exception to this general prediction is the negative relation between pink-footed goose population size and the fraction of that population foraging in agricultural areas. This disparity is likely caused by the population’s spatial distribution, which differs from that of the other species. While barnacle, greylag, and white-fronted geese are known to forage throughout Friesland, pink-footed geese are only observed in Friesland’s south-western part (Koffijberg et al. 2010), where they forage mostly in accommodation area. Increasing pink-footed goose population size may thus result not only in increased numbers in scaring areas, but also in nature areas, explaining why the predicted relationship between pink-footed goose numbers and yield loss is less steep compared to those of the other species. This indicates that the original distribution of geese affects how damage increases with population size.

The variation in goose pressures with changing population sizes may result from changes in foraging behaviour, such as utilizing foraging locations closer to or further from roost-sites, longer or shorter searches as grass lengths at chosen foraging patches are insufficient or clipped at the right height, and increased or decreased scaring as flocks venture into scaring areas more or less frequently. All these emergent behavioural changes result in changes in flight times. Since flight is an energetically costly behaviour (Norberg 1996; Butler et al. 2000; Bishop et al. 2015), this causes a change in energetic requirements, and hence in foraging time and subsequent yield loss. When considering management through scaring and accommodation, careful consideration should be given to how these practices may affect flight times, for example by careful selection of accommodation areas close to roost-sites. When accommodation areas are inadequate, increased energy expenditure due to scaring may result in decreased body condition (Belanger and Bedard 1990; Béchet et al. 2004) and even population decline (Jensen et al. 2008).

The presence and abundance of different goose species may affect species’ spatial distributions and thus their impact on agricultural damages, both through competition for resources and facilitation (van der Wal et al. 2000; Arsenault and Owen-Smith 2002; Stahl et al. 2006). Such patterns also emerged in our model simulations, where species interacted by shortening the grass on visited grassland patches. The fraction of barnacle geese (the smallest of our four study species) foraging in scaring areas increased with its own population size, but decreased with increasing numbers of greylag, pink-footed, and white-fronted geese. Since barnacle goose weight did not decrease, this indicates facilitation by the other goose species. The optimal sward height for foraging by barnacle geese is lower than that of the other species (Durant 2003); hence, barnacle geese may profit from foraging by greylag, pink-footed, and white-fronted geese. Such facilitation can increase foraging efficiency for barnacle geese and decrease their total goose pressure when population sizes of the larger species increase. This model prediction is in agreement with previous studies where smaller species made use of areas previously foraged by larger grazers (Rees 1990; Heuermann 2007).

Our model predicts a clear effect of barnacle geese on white-fronted geese, where more individuals of the larger species end up on agricultural land, and especially in scaring areas, when the smaller species’ population size increases. This fits with previous findings, where smaller waterfowl cropped the grass too short for the larger species’ large bills, thus outcompeting them (Rees 1990; Heuermann 2007). This forces larger species into scaring areas, where they experience high levels of scaring, and subsequently compensate the additional flying time by foraging more. Together with their larger body mass, this results in more damage per goose.

Effectively, greylag geese are predicted to cause the most yield loss per goose. This result is in line with our expectation, as greylag geese are the largest species with the highest energy requirement and intake rate. Similar to white-fronted geese, resource competition causes large parts of the greylag goose population to move to scaring areas when goose numbers increase, resulting in more scaring events, additional foraging, and more scaring patches affected by greylag geese. Yet, a previous study (Buitendijk et al. 2022) found that greylag geese by themselves did not have a significant effect on yield damage. This study used damage reports of grasslands inside accommodation areas, disregarding yield losses in scaring areas, and suggested that greylag geese may forage mainly outside accommodation areas. Both field observations and our model’s predictions indicate that greylag geese indeed spent much time in scaring areas; more than any of the other species. A follow-up study, akin to that of Buitendijk et al. (2022) but using damage reports from both scaring and accommodation areas, may give more insight into the goose species’ roles in the farmer-goose conflict.

In our model, we disregarded the relation between grass height and grass growth, where grass growth rate is maximized at a certain height (Buitendijk and Nolet 2023). By feeding on grass, geese may reduce grass height to approximate this optimal value, stimulating grass growth and increasing total productivity of grasslands. Geese also return some of the removed nutrients through their droppings (Kear 1963). Yet, grass that has been shortened too much may have a reduced growth rate. The relationship between grass height and growth rate appears to change with the season (Buitendijk and Nolet 2023), causing larger effects of grazing on yield when it occurs later in spring. Further research on this topic is needed, and a subsequent adjustment to the model may provide more insight into the relations between goose species interactions, timing of grazing, and yield loss.

## Conclusion

With increasing goose population sizes, conflicts between farmers and geese are becoming a large concern. To reduce farmer-goose conflict, there needs to be a balance between goose welfare and the economic costs associated with their foraging on agricultural grasslands. To do so, new management regimes should take into account the spatial configuration of accommodation and nature areas in relation to current distributions of roost-sites and foraging locations, as well as the species’ interactions affecting those choices. More research is needed to investigate how different management regimes, presenting different spatial configurations, affect goose foraging behaviour and well-being, interspecific interactions, and all costs involved. While living with nature might be a challenge in areas with many human-wildlife conflicts, effective governance of such areas may appease the contrasting needs of man and nature.

## Supplementary Information

Below is the link to the electronic supplementary material.

Supplementary file1 (PDF 1773 kb)

## Data Availability

The source code of the model (C++) and generated datasets are available upon request.

## Footnotes

1
www.bij12.nl/onderwerp/faunaschade/schadecijfers.
