# Correction: A multi-species model for goose management: Competition and facilitation drive space use of foraging geese

**DOI:** 10.1007/s13280-025-02279-6

**Published:** 2025-11-20

**Authors:** Monique de Jager, Nelleke H. Buitendijk, J. M. Hans Baveco, Menno Hornman, Helmut Kruckenberg, Andrea Kölzsch, Jesper Madsen, Sander Moonen, Kees H. T. Schreven, Bart A. Nolet

**Affiliations:** 1 Quantitative Biodiversity Dynamics, Department of Biology, Utrecht University, Budapestlaan 17, 3584 CD Utrecht, The Netherlands; 2 Department of Animal Ecology, Netherlands Institute of Ecology (NIOO-KNAW), Droevendaalsesteeg 10, 6708 PB Wageningen, The Netherlands; 3 Fauna Management Unit Noord-Holland, Spaarne 13, 2011 CD Haarlem, The Netherlands; 4 Environmental Sciences Group, Wageningen University and Research, Postbus 47, 6700 AA Wageningen, The Netherlands; 5 Dutch Centre For Field Ornithology (Sovon), Toernooiveld 1, 6525 ED Nijmegen, The Netherlands; 6 Institute for Waterbird and Wetlands Research (IWWR) Germany e.V., Am Steigbügel 3, 27283 Verden (Aller), Lower Saxony Germany; 7 Max Planck Institute of Animal Behavior, Am Obstberg 1, 78315 Radolfzell, Germany; 8 Ecology Department, Radboud Institute for Biological and Environmental Sciences (RIBES), Radboud University, Heyendaalseweg 135, 6525 AJ Nijmegen, The Netherlands; 9 Wageningen Environmental Reserach (WEnR), Droevendaalsesteeg 3a, 6708 PB Wageningen, The Netherlands; 10 Institute for Avian Research, An Der Vogelwarte 21, 26386 Wilhelmshaven, Germany; 11 Department of Theoretical and Computational Ecology, Institute for Biodiversity and Ecosystem Dynamics, University of Amsterdam, Science Park 904, 1098 XH Amsterdam, The Netherlands; 12 Department of Ecoscience, Aarhus University, C.F. Møllers Allé 8, 8000 Aarhus C, Denmark

**Correction to: Ambio** 10.1007/s13280-025-02206-9

In the original version of this article, the co-author names Jesper Madsen and Kees H. T. Schreven and their biographies were missing due to an oversight of the corresponding author. The correct version of the author group and the missing author biographies are now provided.

Also the e-mail address of the author Nelleke H. Buitendijk and the affiliation information along with e-mail address of the author Bart A. Nolet are updated.

The original article has been corrected.

