# Supplementary material for: A multi-species model for goose management: Competition and facilitation drive space use of foraging geese
Source: Ambio. 2025 Jun 12;55(2):450–64. doi: 10.1007/s13280-025-02206-9 (PMC12779887; doi:10.1007/s13280-025-02206-9)
Supplement: Supplementary file 1 — Supplementary file1 (PDF 1773 kb) [file 13280_2025_2206_MOESM1_ESM.pdf]

Ambio

Supplementary Information

This supplementary information has not been peer reviewed.

Title: **A multi-species model for goose management: competition and facilitation drive space use of foraging geese**

Contents: **Page:**

Contents	Page
Supplementary methods A	2
Table S1	10
Figure S1	11
Figure S2	12
Figure S3	13
Figure S4	14
Figure S5	15
Figure S6	16
Figure S7	17
Figure S8	18
Figure S9	19
Figure S10	20
Figure S11	21

Supplementary methods A: full account of the multi-species individual based model

1. Purpose

The purpose of the model is to examine how four goose species with varying population sizes (barnacle geese (*Branta leucopsis*), greylag geese (*Anser anser*), pink-footed geese (*Anser brachyrhynchus*), and (greater) white-fronted geese (*Anser albifrons*)) affect each other's spatial distribution, assuming management with scaring and accommodation areas. We subsequently investigate the emergent interaction effects of these species on yield loss, appraisal costs, and scaring costs.

2. Entities, state variables, and scales

Flocks of 1,000 barnacle geese, 100 greylag geese, 200 pink-footed geese, and 400 white-fronted geese are the agents in the model. Per species, all flocks are similar, differing in their starting location only. Flocks move around on patches representing the roost sites, natural grasslands, and agricultural grasslands of the province Fryslân in the Netherlands. Flocks keep track of the location and grass height at the last 100 foraging patch visits and the location of their last visited roost site. The flocks' activities are limited to flying, roosting, and foraging.

The spatial scale is set such that each patch represents 100 x 100 m (= 1ha) of Fryslân. The total extent of the modelled area is 70 x 70 km (= 700 x 700 patches of 1 ha). Each patch contains a patch type (-1 for roost, 0 for other, 1 for agricultural grassland in scaring area, 2 for agricultural grassland in accommodation area, and 3 for semi-natural grassland in nature area). Patch types 1, 2, and 3 also contain the variable grass height, and record the number of flock-hours that flocks have spent on the patch. The temporal grain of the model is 1 hour and the temporal extent is set to a single season of 195 days (= 4680 time-steps).

3. Process overview and scheduling

Model setup is discussed in section 5: Initialization.

Each time step, a flock follows the decision tree illustrated in Fig. S2. During daylight hours, flocks move to a selected roost, if maximum weight (median goose weight estimated from field studies for that date plus a maximal additional weight; Table 1; Fig. S3) was reached. If not, they continue foraging. Foraging patches are selected based on memory or at random, depending on memory decay rate (λ), maximum probability to forage on memory (P_{maxM}), and memorized grass heights. To limit memory size, the oldest memory is replaced by the newest one, keeping a constant memory size of 100 locations and their grass heights. A memorized patch is selected based on memory age, the expected energy gain, and the energy required to move there, making it likely that the same patch is used in multiple consecutive time steps. When foraging randomly, a patch is selected using a composite random walk, consisting of a Brownian (exponential distribution) and a truncated Lévy (bounded Pareto distribution) walk.

During flight to the selected patch, the flock may join a patch with other foraging geese of the same species, depending on the number of geese already present. After arriving at a patch, flocks choose whether to forage there, or move again, depending on grass height and maximum probability to forage at a patch (P_{maxF}). Additionally, flocks move if a disturbance occurs, which is more likely in scaring areas. After a flock is disturbed, memorized grass height for the patch is set to zero, making a return in subsequent time steps less likely.

As geese have been observed to forage at night (Lameris et al., 2021), we included this possibility when the flock has a lower than expected weight (Fig. S3), provided they can rest for at least eight hours, and sufficient moonlight is available. Alternatively, geese roost from sunset to sunrise. Flocks return to their previous roost-site if located within 10 km; otherwise a random roost-site is chosen, weighed by the distance to the current location.

4. Design concepts

a. *Basic principles*

Flocks are attracted to other flocks of the same species and prefer to forage on grass which height maximizes species-specific intake rate. Disturbances reset a flock's memory of grass height at the location of the disturbance to zero. Foraging and movement behaviour influences goose energy intake and expenditure. A flock dies when the weight of its geese falls below a species-specific minimum weight value (Table 1). Foraging behaviour also reduces grass height at grazed patches. Each day the grass grows, following Monteith (Monteith, 1977), depending on temperature and solar radiation.

b. *Emergence*

Through the interplay between foraging, grass depletion and growth, and scaring probability, (spatial) distributions of flocks, yield loss, and scaring activity emerge.

c. *Sensing*

Flocks are aware of other flocks at patches along their movement path, grass height of the selected patch, and their location in relation to the previous roost site.

d. *Interaction*

Flocks are attracted to other flocks. When foraging together, information on memorized grass height is exchanged between flocks.

e. *Stochasticity*

Both the initial distribution of flocks and the specific movement decisions modelled have a strong stochastic component. Multiple simulations are needed before conclusions can be drawn.

f. *Collectives*

Although flocks are attracted to other flocks of the same species along their flyway, collective behaviour of flocks is not explicitly modelled. Collective movement patterns could emerge.

g. *Observation*

The main observations are the yield loss per area type, average number of flock-hours per area type, average goose weight, goose survival rate, fraction of patches affected by flocks per area type, and the number of scaring events that occurred during the simulation.

5. Initialization

Flock initialization – We initialized the spatial distribution of our modelled geese based on roost count estimates collected in 2019 by the Dutch Centre for Field Ornithology (Sovon) (Hornman et al. 2021a). Sovon also provided monthly daytime goose counts covering the whole province. These were used to determine the total number of geese present in each month (Fig. S1), which changes with migratory arrival and departure (with a peak in January-February) (Hornman et al. 2021a). Goose initial weight is species-specific and given in Table 1.

Patch initialization – We used ‘Basisregistratie gewaspercelen’ to determine which patches are grassland. These were then divided into nature area (using ‘natuurbeheerplannen 2021’-data, 12,546 patches), accommodation area (‘ganzenfoerageergebied 2021’-data, 15,533 patches) and scaring areas (remaining grassland patches, 139,324 patches). Non-grassland patches were divided into roost-sites (7,881 patches) and other. Using hourly GPS-points from tracked geese, we defined roost-sites as locations within a 1km area that were visited during at least four nights (between 0:00-4:00h, local time). Initial grass height at grassland patches was set at 0.094 m.

6. Input data

The patch-input data is described in 5. Initialization – patch initialization.

7. Submodels

Each time step, a flock follows the decision tree illustrated in Fig. S2. Here, a detailed description of the calculations involved is provided.

1. Daylight hour?

The number of daylight hours per day (*D*) was calculated following Forsythe et al. (1995):

$$D = 24 - (24 / \pi) \cdot \arccos(a / b), \qquad \text{(eq. 1)}$$

where

$$a = \sin(6\pi / 180) + \sin(\pi l / 180) \cdot \sin(c), \qquad \text{(eq. 2)}$$

$$b = \cos(\pi l / 180) \cdot \cos(c), \qquad \text{(eq. 3)}$$

and

$$c = \arcsin(0.398 \cdot \cos(0.216 + 2 \cdot \arctan(0.967 \cdot \tan(0.0086 \cdot (J - 186))))). \qquad \text{(eq. 4)}$$

l is the latitude (l = 53 on average for Fryslân) and J is Julian day. Because the model works with discrete time steps of 1 hour, D is rounded. Simulation of a season starts at the first daylight hour of Julian day 334.

2. Daily Energy Intake ≥ Daily Energy Expenditure?

A simulation initiates with goose flocks that have no energy accumulated yet. To obtain energy, the geese need to forage. Energy is lost by moving, foraging, and roosting. At the start of each time step, the net accumulated energy (*NAE*) is compared with the expected energy expenditure of the remaining hours of that day. Expected energy expenditure (EE_{exp}) – assuming that the goose flies to the roost site and stays here for the remainder of the day – is calculated as:

$$EE_{exp} = T_v \cdot VMR + (3600 \cdot (24 - h) - T_v) \cdot RMR, \qquad \text{(eq. 5)}$$

where T_v is the time (in seconds) it takes to fly to the flock’s most recently visited roost patch, *VMR* is the flight metabolic rate (Table 1; Baveco et al. 2011) , *h* is the hour of the day, and *RMR* is the resting metabolic rate (Table 1; Baveco et al. 2011). When the net accumulated energy is lower than the expected energy expenditure, the flock needs to forage; otherwise, it moves to the nearest roost site. The location of this roost may differ from the one used in calculating the expected energy expenditure; to decrease simulation run time, we have chosen to do the calculation with only one roost site in mind (the previously visited one) instead of all possible roost sites. A side effect of this assumption is that geese sometimes forage longer than necessary, as other roost sites may be closer by.

3. At good foraging patch?

Whether a flock stays at a foraging patch depends on the grass height. The probability to remain at the current location (P_{stay}) was calculated as:

$$P_{stay} = P_{maxF} \cdot IIR / IIR_{max}, \quad \text{(eq. 6)}$$

where P_{maxF} is the maximum probability to stay at the current patch ($0 < P_{maxF} \leq 1$), IIR_{max} is the maximum instantaneous intake rate that can be achieved by geese foraging on grasslands (table 1), and IIR is the instantaneous intake rate. The instantaneous intake rate (IIR, in g/s) depends on grass height (L) of the grass at the current patch:

$$IIR_{(L)} = (\alpha\ (\frac{1 + b_2 L}{b_1 L}\ (T_{c0} + c_1 L) + \frac{1}{R_{max}}))^{-1}, \quad \text{(eq. 7)}$$

where α is a factor that is necessary to include alert time (α = 1.05), b_1, b_2, and c_1 are functional response values, T_{c0} is the minimal cropping time, and R_{max} is the maximal chewing rate (Table 1)(Baveco et al. 2011). If a randomly drawn number X ($0 \leq X < 1$) is larger than P_{stay}, the flock moves away in search of another foraging site.

4. Forage on memory?

When a flock needs to move to another foraging site, it can do so by using its memory, or by doing a random search. The probability to forage on memory and move to one of the memorized foraging sites depends on the highest memorized intake rate:

$$P_{mem} = P_{maxM} \cdot max\ IIR_{mem} / IIR_{max}, \quad \text{(eq. 8)}$$

where P_{maxM} is the maximum probability to forage on memory ($0 > P_{maxM} \geq 1$). Using this probability to move to memorized foraging sites, we can calculate the probability that a flock would rather move to a random location instead, as: $P_{random} = 1 - P_{mem}$.

A flock's memory consists of m (m = 100) memorized locations and grass heights. When a randomly drawn number X ($0 \leq X < 1$) is larger than P_{random}, the flock makes a weighted random selection from the memorized foraging locations, based on the expected gain (which depends on flight time and memorized grass height) and the memories' age:

$$W_i = (expected\ MEI_i - EE_i) \cdot (1 - age_i / \lambda), \quad \text{(eq. 9)}$$

where λ is the memory decay rate ($\lambda > 1$), MEI_i is the expected metabolizable energy intake at location i and EE_i is the expected energy expenditure to move to and forage at location i. The expected metabolizable energy intake is calculated as:

$$MEI_i = IIR_{(Li)} \cdot (T_{max} - T_{v,i}) \cdot MEC_i, \quad \text{(eq. 10)}$$

where MEC_i is the metabolic content (MEC = 7300 J/g at improved grassland, MEC = 6700 J/g at semi-natural grasslands; Baveco et al. 2011), T_{max} is the maximum time spent foraging on a patch (T_{max} = 3600 s), and $T_{v,i}$ is the time to fly to the memorized patch i:

$$T_{v\ exp,i} = \sqrt{[(x - x_i)^2 + (y - y_i)^2]} \cdot L_{patch} / V, \quad \text{(eq. 11)}$$

where x,y are the x- and y-coordinates of the flock's current location, x_i,y_i the x- and y-coordinates of the memorized location, L_{patch} is the length of a patch (L_{patch} = 100m), and V is the flight speed (Table 1)(Baveco et al. 2011). The expected energy expenditure was calculated as:

$$EE_i = (T_{max} - T_{v\ exp,i}) \cdot FMR + T_{v\ exp,i} \cdot VMR, \quad \text{(eq. 12)}$$

where *FMR* is the field metabolic rate and *VMR* is the flying metabolic rate (Table 1) (Baveco et al. 2011). Based on the memory weights W_i, a memorized foraging location is randomly chosen.

5. Select random location

When X is smaller than or equal to P_{random}, the flock uses a random search strategy to move to another foraging site. When a flock uses a random search instead of moving to a memorized location, a flight direction is randomly drawn from a uniform distribution (0 – 360 degrees), together with a flight length (L_v), which is randomly drawn from a composite search strategy, which combines a Levy search with Brownian movement. Per species, we created a composite random walk, in which P_x is the probability that a flight length x (in hm) is drawn from the frequency distribution:

$$P_x = P_{BW}e^{-\lambda x} + (1 - P_{BW})(1 - (1 - x_{min}^{\mu-1}x^{1-\mu})/(1 - (\frac{x_{min}}{x_{max}})^{\mu-1})), \quad \text{(eq. 13)}$$

where x_{min} is the minimum flight distance (x_{min} = 1 hm) and x_{max} is the maximum flight length. We used the distances moved per hour by GPS-tracked geese (de Jager et al. 2023) to estimate the parameters μ, λ, x_{max}, and P_{BW} (Table 1). The parameter P_{BW} gives the probability that a flight length is drawn from an exponential distribution rather than a bounded pareto distribution. Parameters per species were estimated by maximum likelihood estimation of the calculated inverse cumulative frequency distribution (ICFD) on the observed ICFD of distances moved per hour. The selected angle and flight length together result in a target patch. A foraging patch will then be selected from the foraging patches nearest to this target patch.

6. Patches with foraging geese on route?

During a flight, it is possible for a flock to stop at an adjacent foraging site. The probability that a flock will join a group of conspecifics foraging on a patch was estimated using the observed number of geese foraging together in monthly goose counts in 2016-2019 (Hornman et al. 2021). We calculated the relative frequency that a flock was foraging in a group of conspecifics consisting of N flocks (Fig. S10), where a flock's size corresponds to the estimated flock size of that species shown in Table 1. Per species, we estimated the coefficient a_1 in the relation between this relative frequency and group size $F_N = (N + 1)^{a_1}$. Similar to the single-species model (de Jager et al. 2023), where we calibrated the coefficient a_2 with group size data, we used a_2 = 0.1 to estimate the probability to join other flocks of the same species as $P_N = a_2(N + 1)^{a_1}$. To keep the model as simple as possible, we assume that the four species do not actively aggregate nor avoid each other, and hence do not model any direct interactive behaviour (only indirect interactive effects from grass consumption).

7. Move to patch

As the flock flies to the chosen foraging patch, it loses energy (flying metabolic rate: *VMR*) and foraging time (flight speed V = 19.0 m/s; Baveco et al. 2011). From field observations, we know that geese do not fly directly to a foraging patch, but rather circle a patch several times before landing. Using GPS-tracking data, we estimated how much time geese spend on flying per distance between the start and end of a flight (see Fig. S6). We assumed that a goose was flying when the recorded velocity exceeded 12 m/s, and we only used consecutive GPS-locations if the time interval was less than or equal to 15 min. We estimated a species' flight duration as

$$T_v = e^{T_{v1}} \cdot \frac{d \cdot L_{patch}}{V}, \quad \text{(eq. 14)}$$

where d is the flight distance (in patch units), L_{patch} the length of a patch (in m), V is the flight speed (in m/s) (Baveco et al. 2011), and T_{v1} a logarithmic sigmoid function that significantly increases the modelled relation between flight distance and duration in all four species:

$$T_{v1} = \frac{10}{1+ c_1 \cdot (d \cdot L_{patch})^{c_2}}, \quad \text{(eq. 15)}$$

where c_1 and c_2 are species-specific coefficients (Table 1).

8. Disturbance?

At all foraging patches, geese are disturbed unintentionally, and at agricultural grasslands in scaring areas, geese can be disturbed intentionally, with probabilities $P_{disturb}$ and $P_{scaring}$, respectively ($0 \leq P \leq 1$). When a randomly drawn number X ($0 \leq X < 1$) is smaller than or equal to P, the patch is disturbed and the flock relocates. Since we did not have detailed data on return times of geese in Fryslân, we did not explicitly model return time. However, we did set a flock's memorized grass height of the disturbed patch to zero. This way, a flock may only return to the patch when foraging randomly instead of on memory, which is more likely to occur if the site is close to a roost. The disturbed patch is memorized as a patch with no energy gain (memorized grass height = 0.0 m). Between management scenarios, the probability of intentional disturbances in scaring areas is set at different values to investigate the effect of disturbances on goose foraging behaviour. A flock is disturbed maximally once an hour.

9. Forage here

If the selected patch is of sufficient quality (see 3.), the flock stays to forage. By foraging at a patch, geese take in energy and decrease grass height. Their net accumulated energy (*NAE*) increases with $MEI_{(L)} - EE_{(L)}$, which depend on grass height L and flight time T_v. Grass height at the foraging patch ($L_{x,y}$) decreases:

$$L_{x,y} = L_{x,y} - (IIR_{(Lx,y)} \cdot (T_{max} - T_v) \cdot N_{flock}) / (d_l \cdot A), \quad \text{(eq. 16)}$$

where T_{max} is the maximum foraging time (= 3600 s), T_v is the flight time (s), N_{flock} is the flock size, d_l is the leaf density (d_l = 1298 g/m^3; Heuermann 2007), and A is the patch area size (A = 10,000 m^2).

10. Roosting

When a flock is going to roost, and is already at a roost site, it will stay there; otherwise, the flock needs to move to a roost site. To substantially decrease simulation time, flocks return to their previous roost site if it is within 10 km distance from the flock. Otherwise, a roost patch is drawn using a weighted random selection (weight = 1/distance to roost patch). The flock flies to the roost patch in a straight line. Flying to the roost patch will decrease a goose's accumulated energy and its time at the roost. At the roost patch, energy is lost in resting. Together with moving to the roost site, the energy expenditure at the roost is deducted from the net accumulated energy (*NAE*) budget:

$$NAE = NAE - (T_v \cdot VMR + (3600 - T_v) \cdot RMR). \quad \text{(eq. 17)}$$

11. Next time step

Once all flocks (in random order) have had their turn in roosting or foraging, we simulate the next hour. Every 24 steps, the grass grows, depending on temperature (no growth below 6˚C) and solar radiation. Following Monteith (Monteith 1977), we calculated the rate of dry matter production D (g m^{-2} h^{-1}) as

$D = (5 + 0.05/G_c)^{-1}$, (eq. 20)

where G_c is the solar radiation, per hour per day. We calculated total solar radiation as:

$$G_c = (\tau_b + \tau_d) \cdot G_{sc} \cdot \left(1 + 0.033 \cdot cos\frac{360n}{365}\right) \cdot cos\theta_z, \quad \text{(eq. 21)}$$

where

$$\tau_b = 0.127 + 0.749 \cdot e^{-0.391}/_{cos\theta_z}, \quad \text{(eq. 22)}$$

$$\tau_d = 0.271 - 0.294 \cdot \tau_b, \quad \text{(eq. 23)}$$

$$cos\theta_z = cos\varphi \cdot cos\delta \cdot cos\omega + sin\varphi \cdot sin\delta. \quad \text{(eq. 24)}$$

G_{sc} is the solar constant (*1376 W/m*^{2}), *n* is the Julian day number, φ is the latitude (53°), δ is the solar declination angle ($\delta = 23.45 \cdot sin(360 \cdot (284 + n) / 365)$), and $\omega = 15 \cdot (t - 12)$, where *t* is the time of day in hours. We calculated *D* per hour as the average over ten 6-minute intervals, and subsequently added up all dry matter production rates of all hours per day to estimate the daily dry matter production. Daily grass growth was calculated as $D/1298\ m\ d^{-1}$ when the average daily temperature was between 6 and 12°C; grass did not grow at temperatures below 6°C and grew twice as fast at temperatures above 12°C. For daily temperatures, we averaged the hourly temperature data per day that was recorded for the years 2015-2019 in Leeuwarden (KNMI).

The current model can be made to be more realistic by using Grass Height Dependent Grass Growth (GHDGG). While we modelled grass growth following Monteith (1977), in reality, grass growth depends on initial grass height and time of year (Buitendijk and Nolet 2023). In the more refined model, for the period after February 14th, we estimated daily grass growth (*dH*, in m) from the empirical two-week growth data of Buitendijk and Nolet (2023), using julian day (*J*) and grass height (*H*, in m) as independent variables (Fig. S11):

$$dH = 0 \vee b_1 H - b_2 H^2,$$

where b_1 = 10 and

$$b_2 = 10 \cdot e^{3.8 - 0.02J}.$$

In the period before February 14th, grass growth is set to zero.

References:

Baveco, J. M., H. Kuipers, and B. A. Nolet. 2011. A large-scale multi-species spatial depletion model for overwintering waterfowl. *ECOLOGICAL MODELLING* 222. Amsterdam: Elsevier Science Bv: 3773–3784. doi:10.1016/j.ecolmodel.2011.09.012.

Buitendijk, N. H., and B. A. Nolet. 2023. Timing and intensity of goose grazing: Implications for grass height and first harvest. *AGRICULTURE ECOSYSTEMS & ENVIRONMENT* 357. Amsterdam: Elsevier: 108681. doi:10.1016/j.agee.2023.108681.

Hornman, M., M. Kavelaars, K. Koffijberg, F. Hustings, E. van Winden, P. van Els, and R. Kleefstra. 2021a. Sovon Ganzen- en Zwanenwerkgroup & Soldaat L. Watervogels in Nederland in 2018/2019. SOVON Vogelonderzoek Nederland.

Hornman, M., M. Kavelaars, K. Koffijberg, F. Hustings, E. Van Winden, P. van Els, and R. Kleefstra. 2021b. Watervogels in Nederland in 2018/2019. Sovon Rapport 2021/01. RWS-rapport BM 21.08 Sovon Vogelonderzoek Nederland. Sovon Ganzen- en Zwanenwerkgroep & Soldaat L.

de Jager, M., N. H. Buitendijk, J. M. Baveco, P. van Els, and B. A. Nolet. 2023. Limiting scaring activities reduces economic costs associated with foraging barnacle geese: Results from an individual-based model. *Journal of Applied Ecology* n/a. doi:10.1111/1365-2664.14461.

Koelzsch, A., G. J. D. M. Mueskens, H. Kruckenberg, P. Glazov, R. Weinzierl, B. A. Nolet, and M. Wikelski. 2016. Towards a new understanding of migration timing: slower spring than autumn migration in geese reflects different decision rules for stopover use and departure. *OIKOS* 125. Hoboken: Wiley: 1496–1507. doi:10.1111/oik.03121.

Monteith, J. 1977. Climate and Efficiency of Crop Production in Britain. *PHILOSOPHICAL TRANSACTIONS OF THE ROYAL SOCIETY B-BIOLOGICAL SCIENCES* 281. London: Royal Soc: 277–294. doi:10.1098/rstb.1977.0140.

Schreven, K. H. T., J. Madsen, and B. A. Nolet. 2024. Effects of capture and GPS-tagging in spring on migration timing and reproduction in Pink-footed geese *Anser brachyrhynchus*. *ANIMAL BIOTELEMETRY* 12. London: Springernature: 10. doi:10.1186/s40317-024-00365-2.

Supplementary Tables and Figures

Supplementary Table 1: *Details on the GPS-transmitters. We only used one GPS point per individual goose per hour, in the winter period (November – May).*

**Species**	**Transmitter type**	**Tracking period**	**N**	**# GPS-points**	**Permit**
Barnacle goose	Ornitela (BP) 25 g (raised solar panel)	2019 – 20	89	138,377	CCD permit 20173788
Greylag goose	MadebyTheo (NB) 35 g	2016 – 20	64	56,942	LAVES AZ 33.19-42502-04-15/1956
Pink-footed goose	Ornitela (NB) 38 g	2019 – 20	32	14,535	See (Schreven et al. 2024)
White-fronted goose	University Konstanz (NB) 35 g; MadebyTheo (NB) 35 g	2016 – '20	149	74,436	See (Koelzsch et al. 2016)

*BP = backback; NB = neckband.

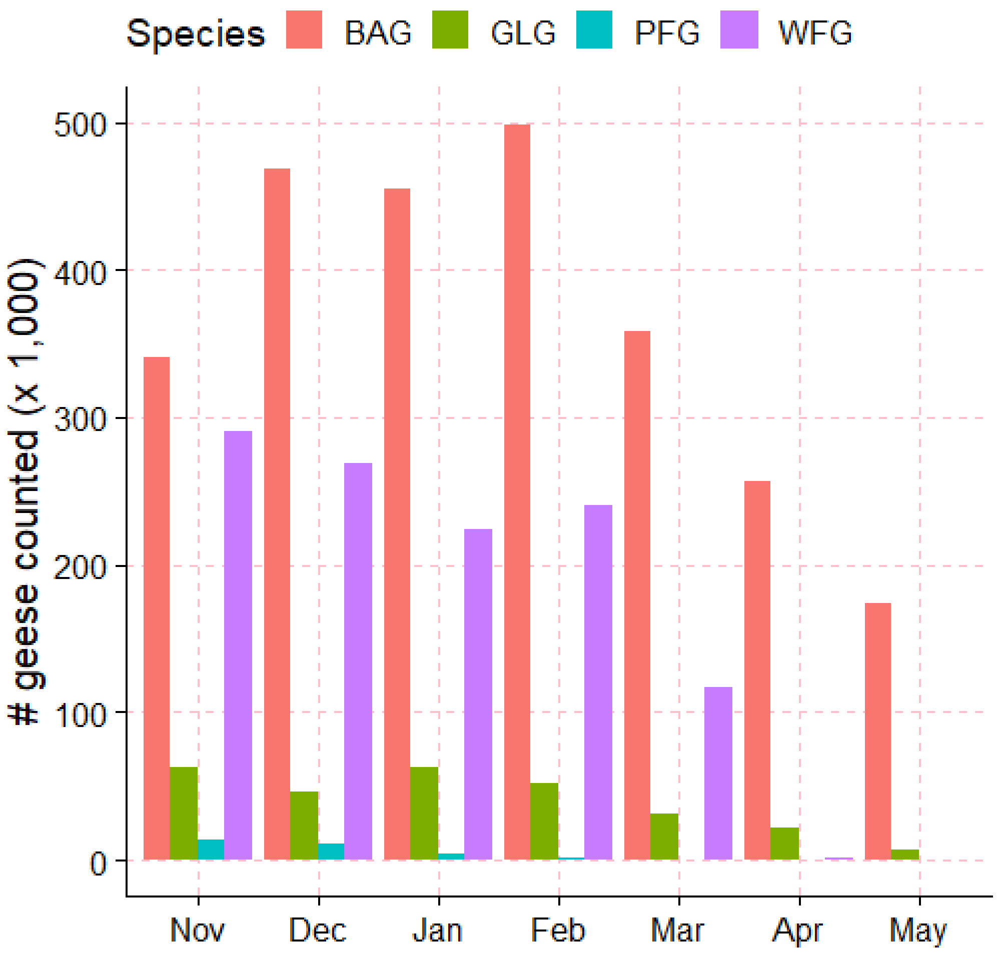

Figure S1: *The maximum counted number of geese per month and species. Barnacle geese are present throughout the simulated period, as are greylag geese. Pink-footed geese have only been seen in November, December, and January. White-fronted geese were also absent in April and May, but present in November-March.*

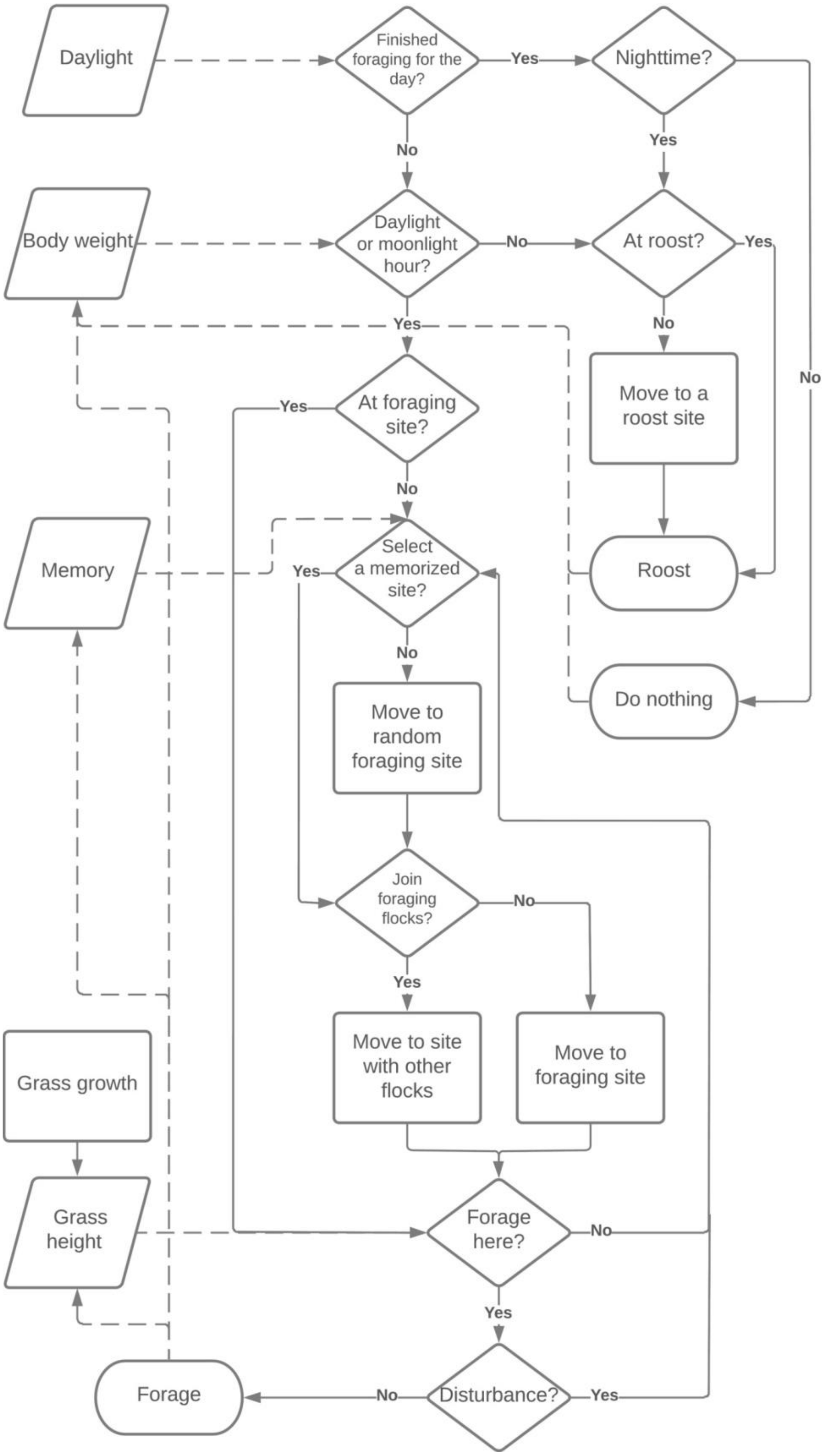

Figure S2: *Schematic overview of the model. The diamond shapes, parallelograms, rectangles and oval shapes indicate decisions, data, processes and endpoints, respectively (from de Jager et al. 2023).*

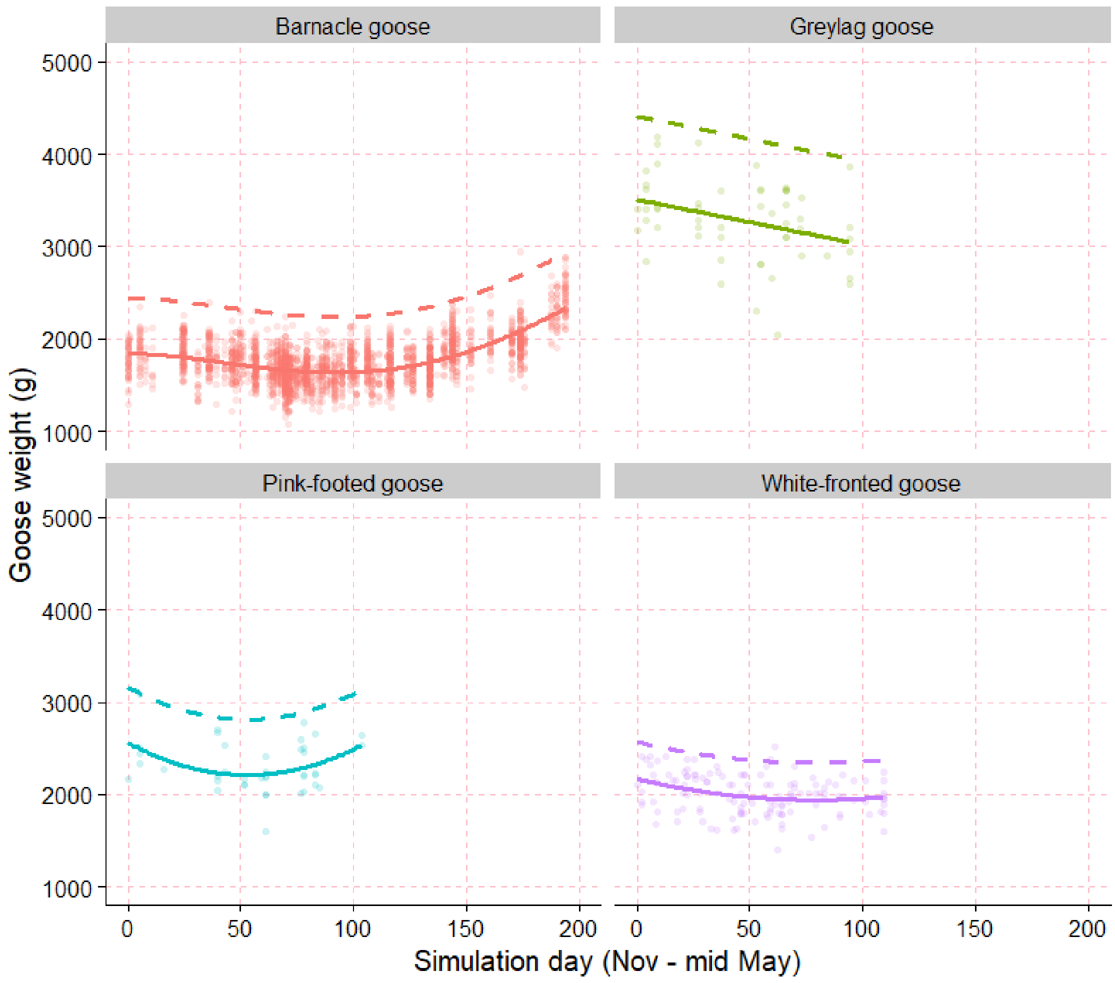

Figure S3: *Recorded weights of captured geese during the period November 1st to May 15th, for the four different species. Solid lines indicate the polynomial function of average goose weight per simulation day. Dashed lines show the maximum goose weight per simulation day, estimated as the average goose weight plus the maximal additional weight (Table 1).*

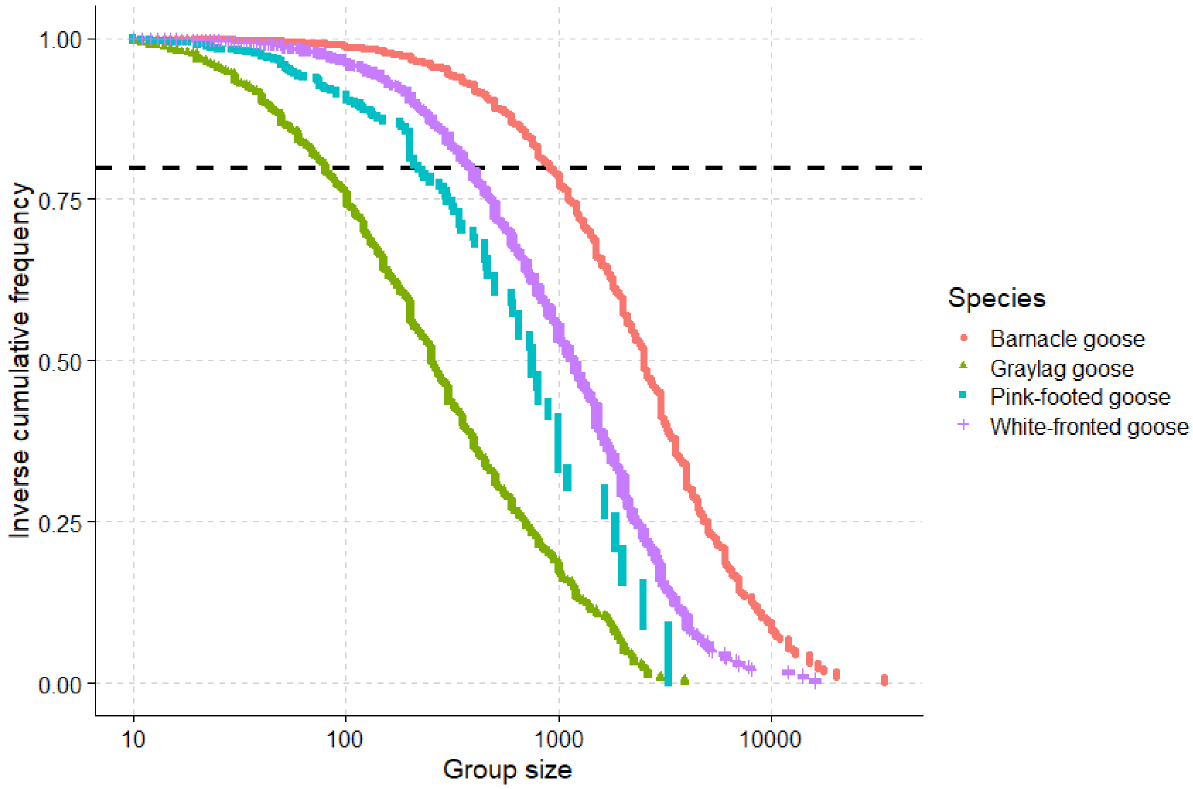

Figure S4: *Inverse cumulative frequency distributions of group sizes of the four goose species. The black dashed line indicates the 20-percentile on which the flock sizes in the model are based.*

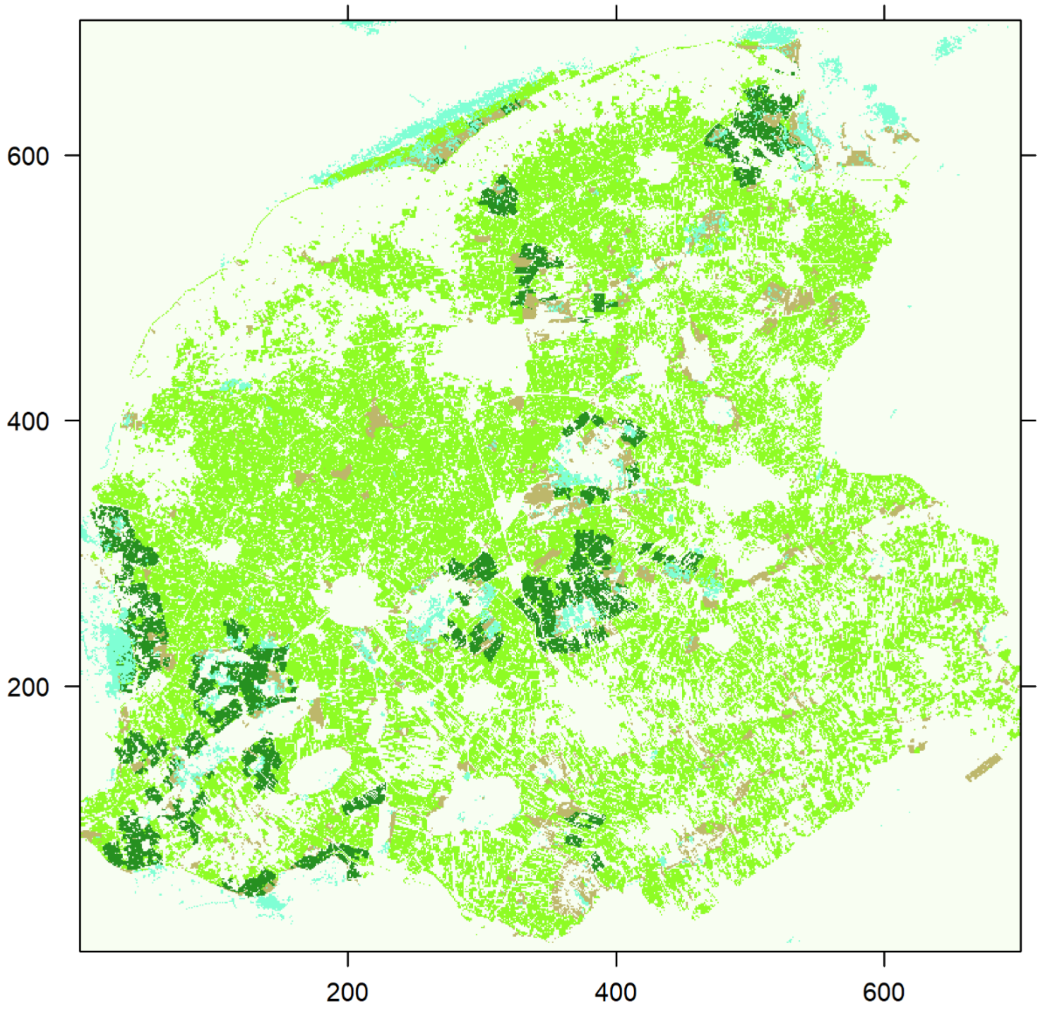

Figure S5: *The simulated landscape, based on the province of Friesland, the Netherlands. Blue areas indicate roost areas, light-green the scaring areas, dark-green the accommodation areas, and taupe the nature areas.*

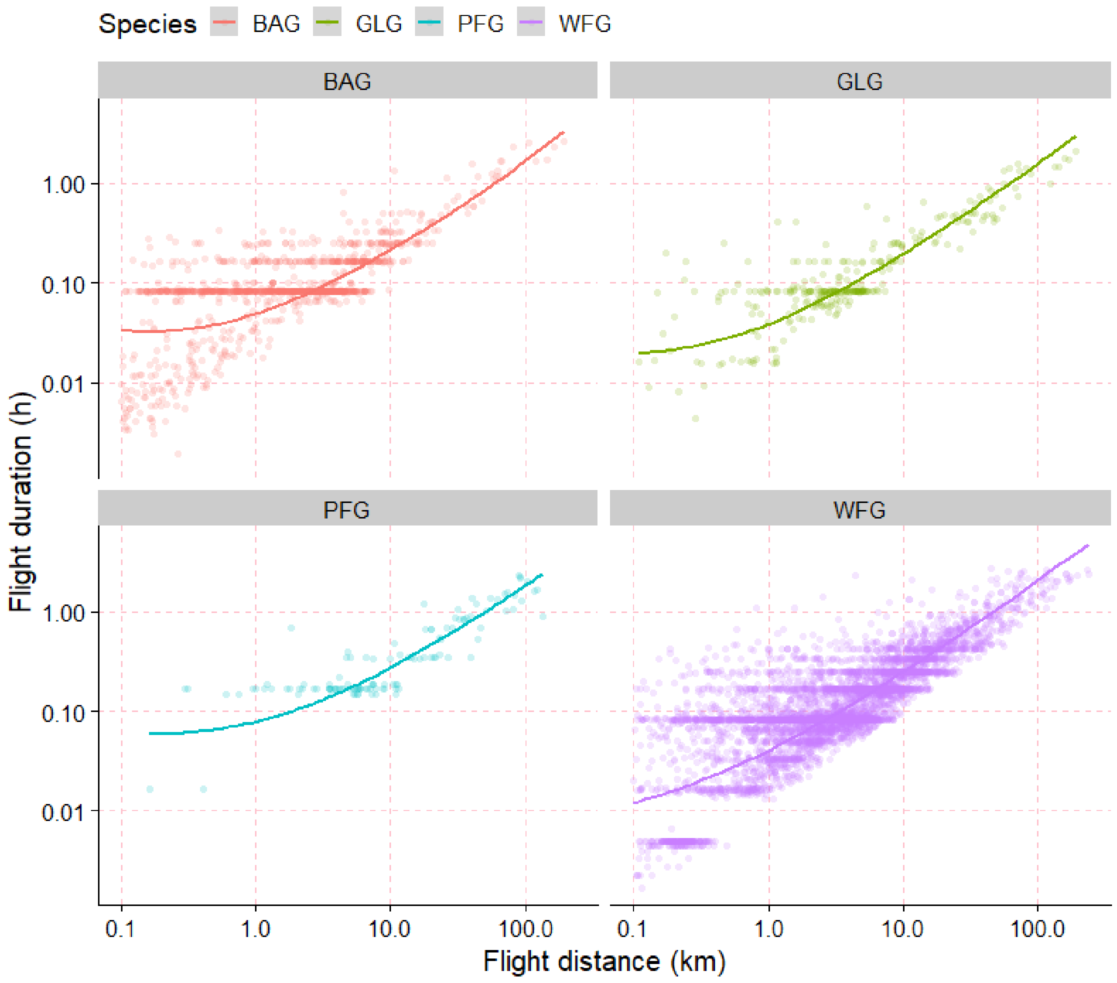

Figure S6: *Relationship between the duration and length of a flight. Points indicate data from GPS tracks, lines indicate the time spend flying calculated with eq. 1. For species abbreviations, see Table 1. Note that the axes are on a logarithmic scale for better visualisation.*

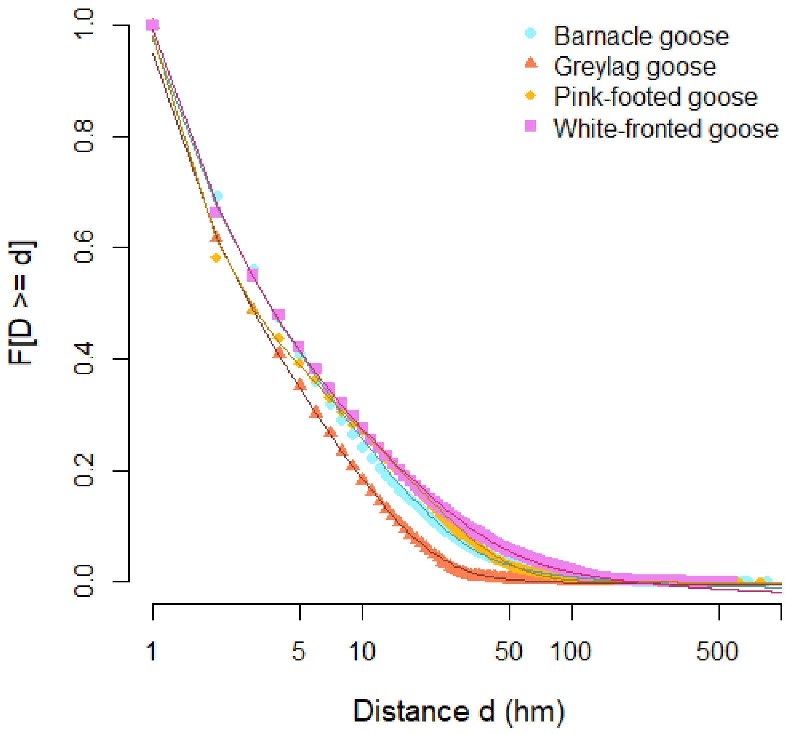

Figure S7: *Inverse cumulative frequency distributions of the distances moved per hour by barnacle, greylag, pink-footed, and white-fronted geese. Lines indicate the fitted composite random walks.*

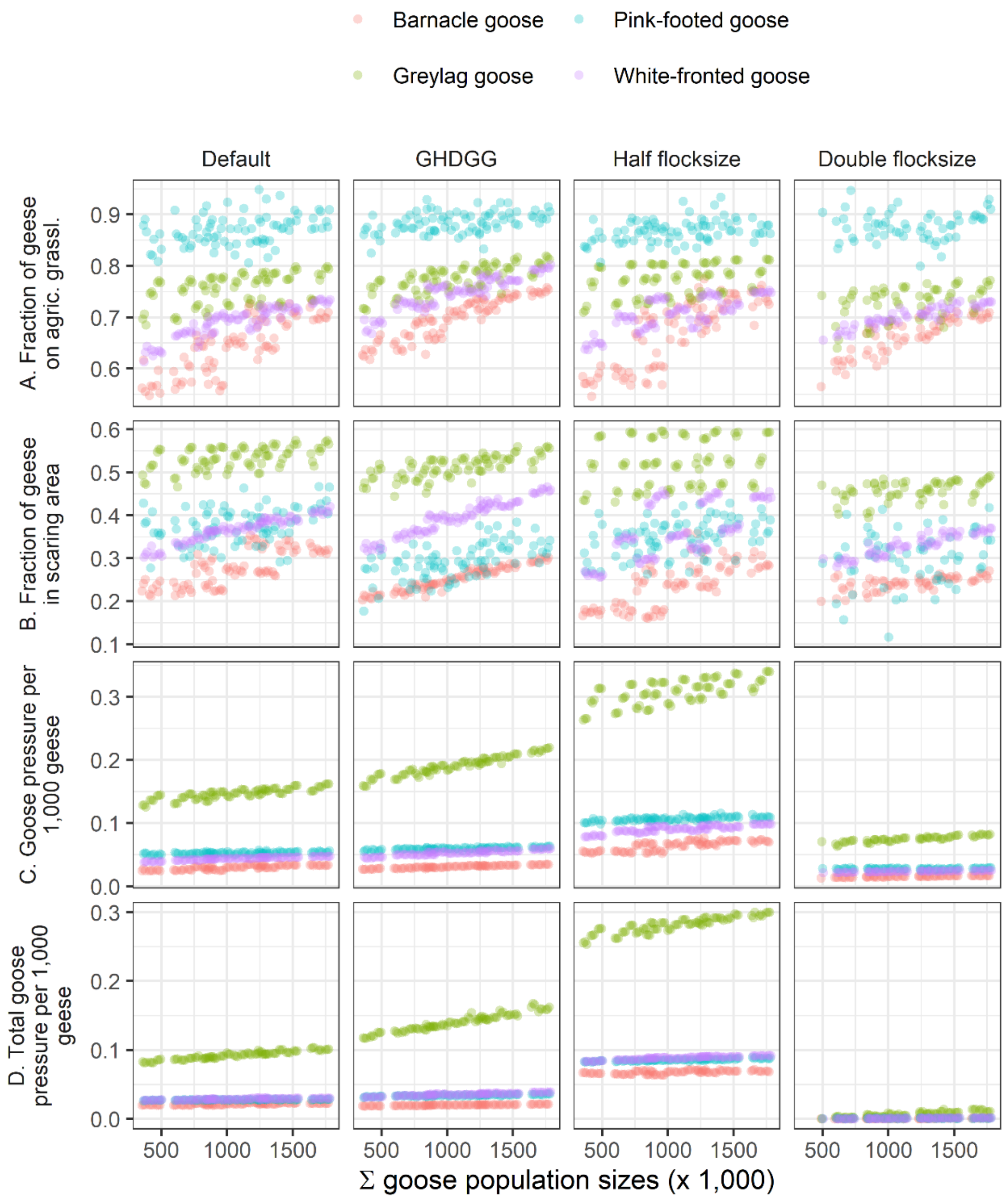

Figure S8: *Results shown for 81 simulations with (1st column) the model as we used it for all other simulations in the manuscript, (2nd column) the model containing grass height dependent grass growth (GHDGG), (3rd column) the model with half sized flocks (flock sizes: BAG = 500, GLG = 50, PFG = 100, and WFG = 200 geese), and (4th column) the model with doubled flock sizes (flock sizes: BAG = 2000, GLG = 200, PFG = 400, and WFG = 800 geese). Figure panels show (A) the fraction of geese of the focal goose species (indicated by colour) foraging on agricultural grassland rather than in nature area, (B) the fraction of geese of the focal species foraging in the scaring area, (C) average goose pressure per 1,000 geese of the focal species (in goose h ha^{-1} day^{-1}), and (D) total goose pressure per 1,000 geese of the focal species (also in goose h ha^{-1} day^{-1}) in relation to the sum of all four goose population sizes per simulation. Goose pressure in C and D is calculated for agricultural grassland only.*

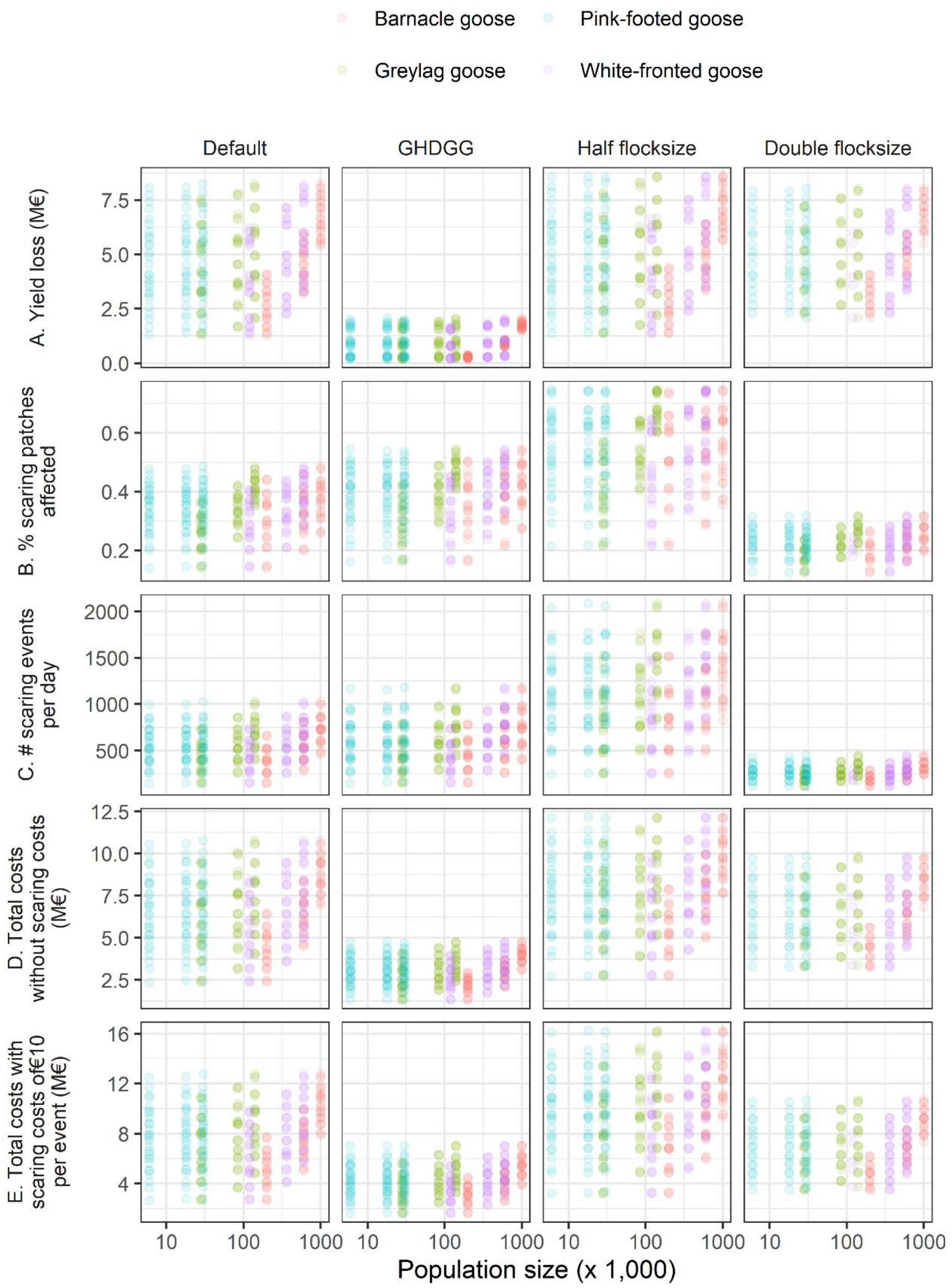

Figure S9: *Results shown for 81 simulations with (1st column) the model as we used it for all other simulations in the manuscript, (2nd column) the model containing grass height dependent grass growth (GHDGG), (3rd column) the model with half sized flocks (flock sizes: BAG = 500, GLG = 50, PFG = 100, and WFG = 200 geese), and (4th column) the model with doubled flock sizes (flock sizes: BAG = 2000, GLG = 200, PFG = 400, and WFG = 800 geese). (A) Yield loss in M€, (B) percentage of agricultural patches affected by geese, (C) the number of scaring events per day, (D) total costs without scaring costs and (E) total costs with scaring costs of €10 per scaring event, per species' population size. Colours indicate the four different goose species.*

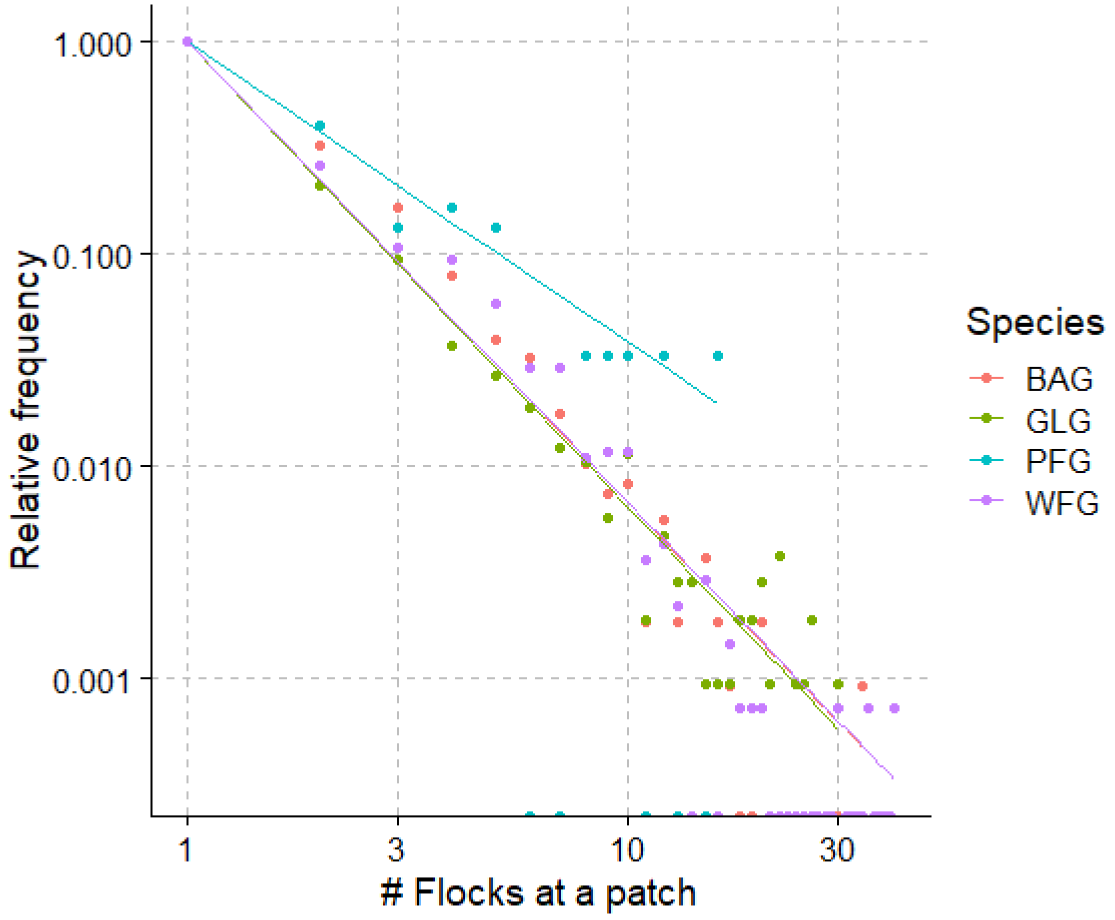

Figure S10: *The relative frequencies of the number of flocks (of sizes 1000, 100, 200, and 400 individuals, for BAG, GLG, PFG and WFG, respectively) observed aggregated together during monthly counts* (Hornman et al. 2021b). *For species abbreviations, see Table 1.*

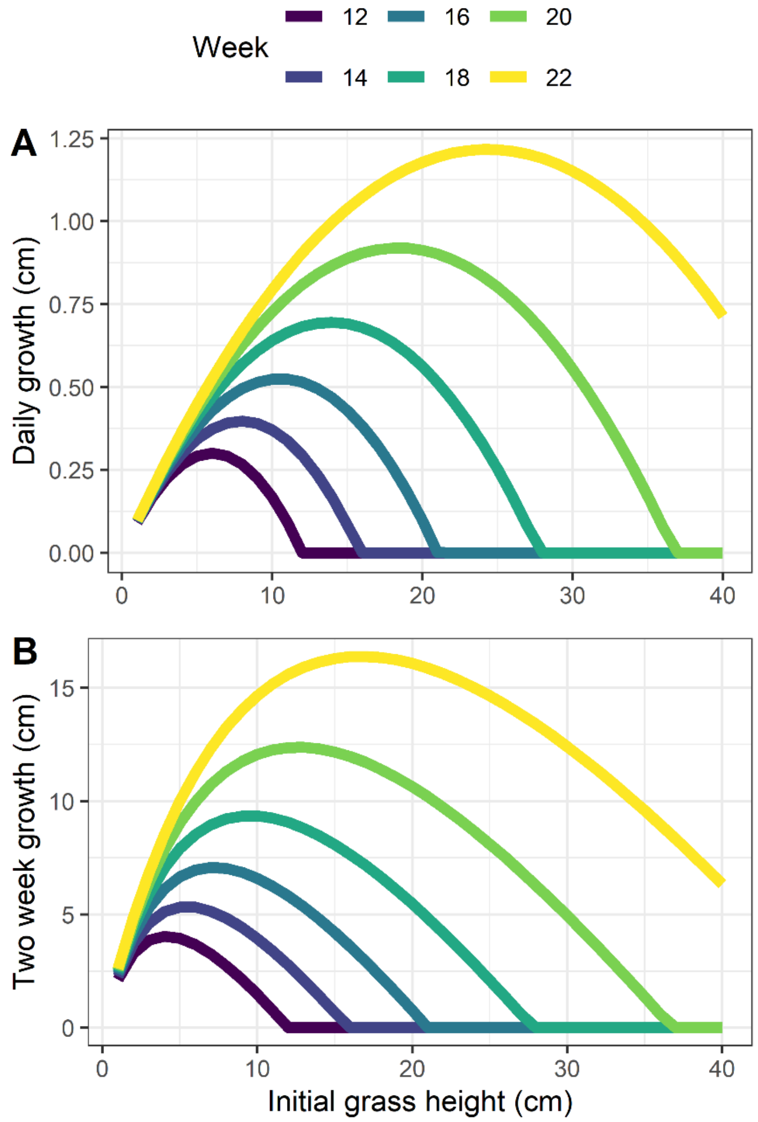

Figure S11: *Grass growth dependent on day number and grass height. Parameters were estimated to resemble the empirical two week growth data of Buitendijk and Nolet (2023). (A) shows daily grass growth and (B) grass growth in two weeks, given the initial grass height (all in cm). Colors indicate growth curves in different weeks of the year, similar to Buitendijk and Nolet (2023).*
